# Global, regional, and national burden and quality of care index in children and adolescents: A systematic analysis for the global burden of disease study 1990–2017

**DOI:** 10.1371/journal.pone.0267596

**Published:** 2022-04-26

**Authors:** Melika Hanifiha, Ali Ghanbari, Mohammad Keykhaei, Sahar Saeedi Moghaddam, Negar Rezaei, Maryam Pasha Zanous, Moein Yoosefi, Erfan Ghasemi, Nazila Rezaei, Sarvenaz Shahin, Mohammad-Mahdi Rashidi, Azin Ghamari, Rosa Haghshenas, Farzad Kompani, Farshad Farzadfar

**Affiliations:** 1 Non-Communicable Diseases Research Center, Endocrinology and Metabolism Population Sciences Institute, Tehran University of Medical Sciences, Tehran, Iran; 2 Feinberg Cardiovascular and Renal Research Institute, Northwestern University, School of Medicine, Chicago, IL, United States of America; 3 Endocrinology and Metabolism Research Center, Endocrinology and Metabolism Clinical Sciences Institute, Tehran University of Medical Sciences, Tehran, Iran; 4 Division of Hematology and Oncology, Children’s Medical Center, Pediatrics Center of Excellence, Tehran University of Medical Sciences, Tehran, Iran; Seoul National University College of Medicine, REPUBLIC OF KOREA

## Abstract

**Purpose:**

To express a global view of care quality in major causes of mortality and morbidity in children and adolescences

**Methods:**

We used primary epidemiologic indicators from the Global Burden of Disease 1990–2017 database. We have created four secondary indices from six primary indices in order to assess the care quality parameters. We conducted a principal component analysis on incidence, prevalence, mortality, Years of Life Lost (YLLs), Years Lived with Disability (YLDs), and Disability Adjusted Life Years (DALYs) to create an index presented by quality-of-care index (QCI) to compare different countries.

**Results:**

The global QCI scores of respiratory infection, enteric infection, leukemia, foreign body aspiration, asthma, epilepsy, diabetes mellitus, dermatitis, road injury, and neonatal disorders have improved remarkably. These causes showed equal distribution of qualified care for both sexes. The global trend of QCI score for mental health showed a steady pattern during the same time and disparities favoring females was evident. The quality of care for these causes was notably higher in developed areas.

**Conclusions:**

The global QCI revealed a universal growth in major causes of death and morbidity in <20y during 28 years. Quality of care is an associate of the level of country’s development. Despite effective interventions, inequities still remain. Implementation of policies to invest in quality improvement and inequality elimination is needed.

## Introduction

Health of children and adolescences is important not only as a human right issue, but as an important determinant of development and sustainability. Investments in children health contribute to social security and economic growth [1]. This special attention is clearly seen in Millennium Development Goals (MDG) [2] and Sustainable Development Goals (SDGs) [3], which are among the most important global policies. Furthermore, based on the critical essence of attaining SDGs related to children and adolescences, a specific roadmap has been created to prioritize the health strategies for these age groups globally [4]. Drawing from the World Health Organization (WHO), quality of care is defined as a successful scope of a health services, resulting in the improvement of desired health outcomes. Fulfillment of this success depends on health care effectiveness, efficiency, accessibility, acceptability, equitability and safety [5]. Following this important issue, publications were released specifically on improving the quality of care for children and young adolescents [6, 7]. Despite all these attentions and a decrease in the number of death rates in children and adolescents during recent decades, several preventable causes still lead to premature death and morbidity worldwide [8]. During a period of 28 years, regardless of implementing comprehensive programs to control the burden of respiratory and enteric infections, they are still among top ten leading causes of disability-adjusted life years (DALYs) [8]. On the other hand, non-communicable diseases are currently a global challenge [9] even in this age group [8]. Investment in development of cost-effective interventions to target the quality improvement and prevention is crucial to decrease burden of diseases in this influential age group [10].

Previous studies on this issue were commonly descriptive [8], and all age groups or all causes were included [11, 12]. We found that a global study that demonstrates status of child and adolescent quality of care in different countries is lacking. Studies demonstrate that overall measures of mortality and morbidity has improved between 1990 and 2017 [9]. However, inequality between developing and developed countries persists owing to unequal growth [8]. In contrast to the trend of changes in mortality and morbidity due to communicable, neonatal, maternal and nutritional diseases in children and adolescents, improvements in non-communicable disease and injuries were less significant during these years [8]. Ten Leading causes of DALYs in population of under 20y in 2017 were as follows: neonatal disorders, lower respiratory infection, diarrhea, congenital disorders, malaria, meningitis, road injury, iron deficiency, protein-energy malnutrition, and HIV [8]. Dermatitis and mental health disorders were among top 10 leading causes of DALYs in high and high-middle Socio-demographic Index (SDI) [8].

In this study we aim to express a global view of quality care in major causes of mortality and morbidity in children and adolescences. In order to evince quality of care and early detection status and trends of different SDI quintiles in recent decades, we created a novel index by the name of quality-of-care index (QCI). Among the top leading causes of DALYS [8] and challenging non-communicable causes, we chose 11 causes to magnitude the situation in different 3 categories [8, 13–15]. For communicable disease: respiratory infections, enteric infections; for non-communicable disease: mental health disorders, asthma, neonatal disorders, diabetes mellitus (DM), idiopathic epilepsy, leukemia and dermatitis, for injuries: foreign body and road injuries.

## Materials and methods

### Overview and data resources

We used the Global Burden of Disease (GBD) data from 1990 to 2017, which is available in GBD compare in Institute for Health Metrics and Evaluation (IHME) website in causes section as: A.2 (Respiratory infection and tuberculosis), A.3 (Enteric infection), A.6.2 (Neonatal disorders), B.1.28 (Leukemia), B.3.3 (Asthma), B.5.3 (Idiopathic epilepsy), B.6 (Mental disorders), B.8.1 (Diabetes mellitus), B.9.1 (Dermatitis), C.1.1 (Road injury), C.2.8 (Foreign body) [16].

The data this study was secondary and no individual data is used. The confidentiality of the data is preserved.

### Quality of care index

We created four secondary indices from six primary indices, in order to assess the quality of care parameters [17]. These indicators are Mortality to Incidence Ratio (MIR), DALYs to Prevalence Ratio, Prevalence to Incidence Ratio, and years of life lost (YLLs) to years lived with disability (YLDs) Ratio. We chose 11 important causes of mortality and morbidity in children and adolescents.

In order to summarize all of these indicators, Principal Component Analysis (PCA) was performed. The first component extracted from PCA was considered as the QCI, which is scaled into 0 to 100 range, with higher scores representing better quality of care. We calculated QCI and SDI quintiles on global scale. SDI is a measure of socio-demographic development in GBD studies, computed by a composite average of the rankings based on average income per person, educational attainment, and total fertility rate (TFR). Classifications of SDI are high, high-middle, middle, low-middle, and low quantiles. It is important to know calculated QCI for each scale, is a compile of 28-year data of that scale. In order to find outliers, 6 sigma approach is used. Mean and standard deviation of indicators are calculated and measures out of μ-3σ, μ+3σ considered as outliers.

### Age and gender disparity

Age classification was in seven groups: early neonatal (0-7d), late neonatal (7-28d), post neonatal (1-12m), 1-4y, 5-9y, 10-14y, 15-19y. The QCI is calculated for each age group, sex, cause, and year in global scale, SDI quintiles, and 195 countries and trends have been depicted disparity of care in different ages.

To calculate the gender disparity, we made gender disparity ratio (GDR), which is QCI score in females divided by QCI score in males.


GDR=(QCI‐F)/(QCI‐M)

Then we calculated this ratio on global scale, SDI quintiles, and all countries. Ratios near one demonstrate the least disparity between two sexes. Values higher or lower than one shows inequality favoring one sex.

### Statistical analysis

Primary indicators are reported with a 95% uncertainty interval (UI). Estimation and trend of changes were significant when UIs during time do not overlap. All the statistical analyses, plots and numbers created in this study were performed by R v3.6.1 and RStudio v1.0.136 [18].

### QCI validity analysis

We have evaluated the correlation between the QCI and Healthcare Access and Quality Index (HAQI) [11] by applying a mixed effect model of QCI as a dependent variable and inpatient health care utilization, outpatient health care utilization, cause-specific death, prevalence, and attributed death to all risk factor as independent variables and considering countries as random effects. The Pearson correlation coefficients between the predicted values with the HAQI were mostly more than 0.70 (S1 Table in S1 File).

## Results

**Fig 1:**
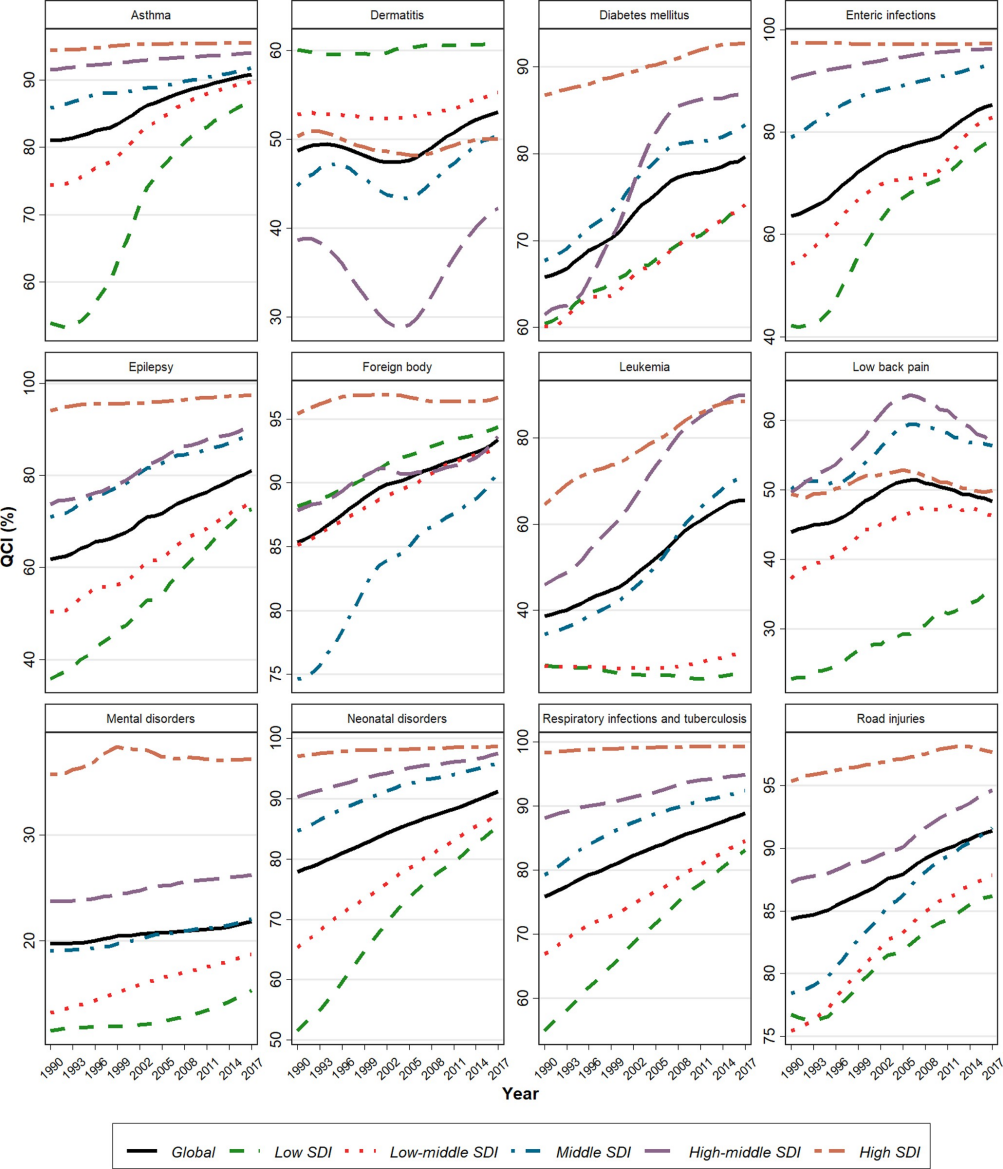
Map of Percent change in deaths rate due to all causes in under 20y population between 1990 and 2017. (Republished from https://www.openstreetmap.org/under a CC BY license, with permission from https://www.openstreetmap.org/copyright, original copyright 2020).

**Fig 2:**
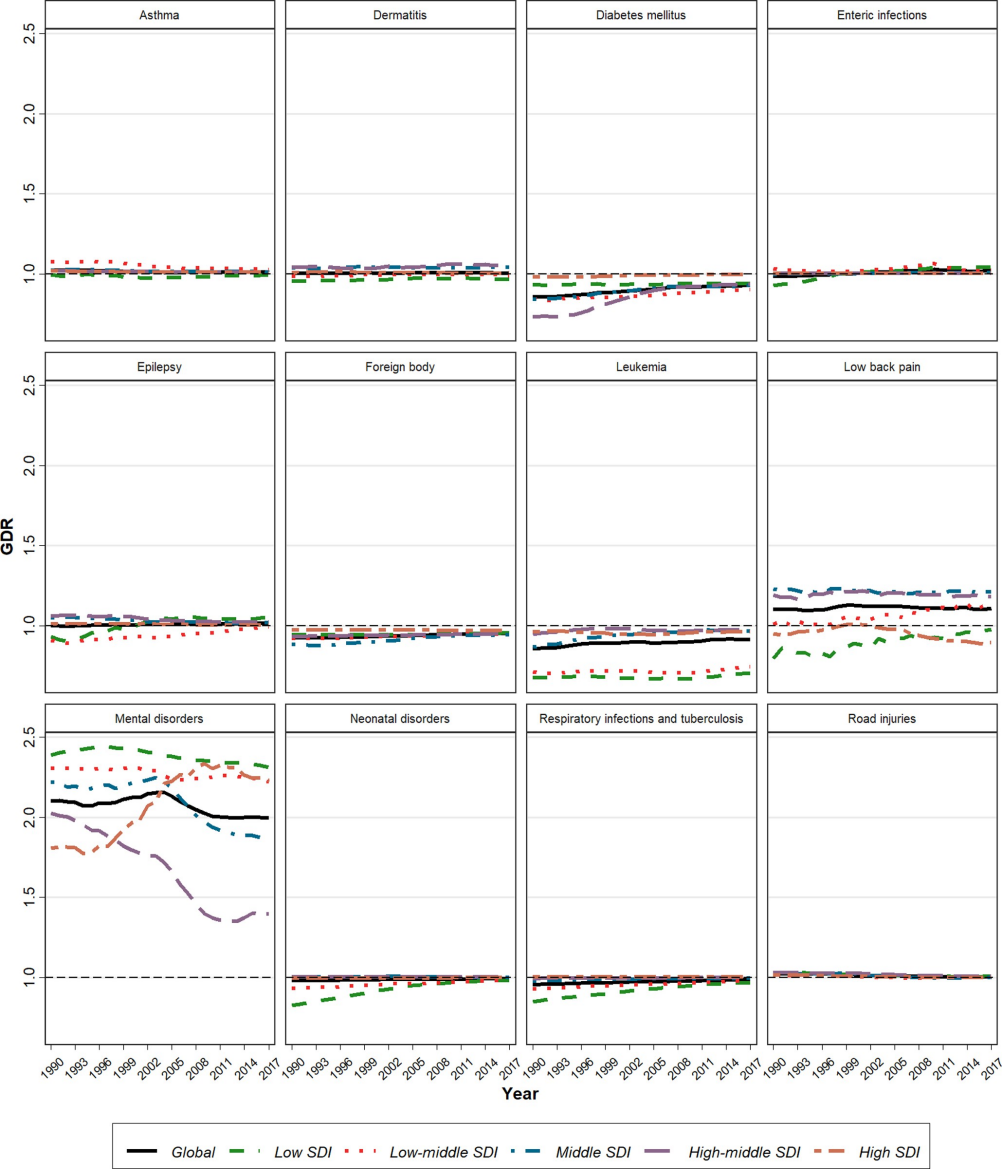
Map of percent change in DALYs rate due to all causes in under 20y population between 1990 and 2017. (Republished from https://www.openstreetmap.org/ under a CC BY license, with permission from https://www.openstreetmap.org/copyright, original copyright 2020).

Global death rate in under 20y population was 255.91 (248.10, 264.78) per 100,000 in 2017, which decreased near 50% between 1990 and 2017 (Fig 1). Total DALY in this age group was 27319.50 (25648.57, 29179.21) per 100,000 in 2017 and it declined 46% during these years (Fig 2). 28.4% of all ages global DALY in 2017, goes for population of under 20y. 53.5% of DALY in this age group belongs to males. 20% of DALY in under 20y comes from YLD. Early neonate age group had the highest burden (23%), then by order post neonatal (20.7%), 1-4y (20.3%), 15-19y (11.4%), 5-9y (9.3%), 10–14 (8.6%) and late neonate age group had the lowest burden (6.3%).

### Neonatal disorders

**Fig 3:**
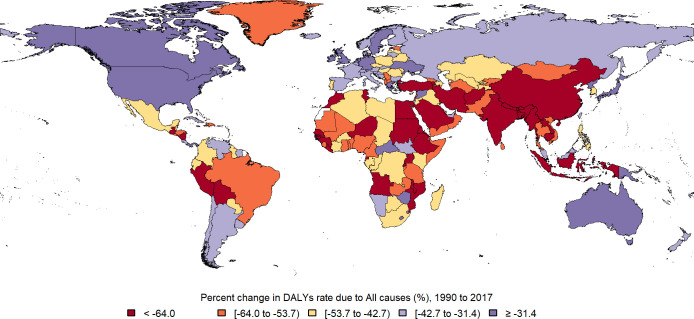
Time trend of Quality-of-Care Index (QCI) between 1990 and 2017.

**Fig 4:**
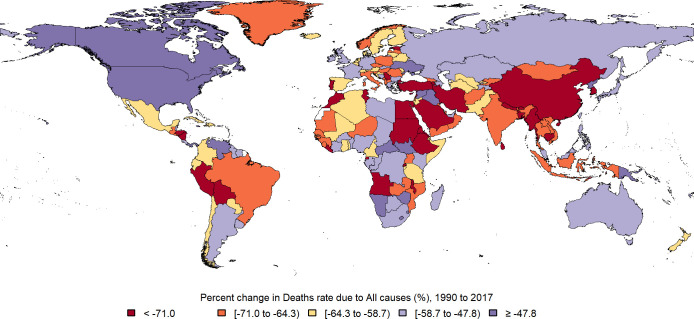
Time trend of Gender Disparity Ratio (GDR) from 1990 to 2017.

Global death rates and DALY decreased in <20y population between 1990–2017 (Table 1, S42 Fig in S2 File). 23.9% of DALY in under 20y population belongs to neonatal disorders. Estimated global QCI score is 91.2. Estimation of QCI scores in different SDI quintiles is 98.6, 97.5, 95.9, 87.4, and 85.3 for high, high-middle, middle, low-middle, and low SDI regions respectively. The highest score belongs to Singapore, and the lowest is for Mali (S2 Table in S1 File, S31 Fig in S2 File). Inspection of QCI scores reveals a growing pattern over a period of 28-year globally and in all SDI quantiles, with significant changes in low-SDI quintiles (Figs 1, 3). In 1990, quality of care was favoring males in low SDI quantiles, but over the time, all SDI quintiles represented an equal condition of care between two sexes, as GDR scores in all ages narrowed just near one (Fig 4).

**Table 1:** Incidence, prevalence, DALY and QCI score in 2017, and its percent changes between 1990 and 2017 in 11 causes.

Cause	Incidence		Prevalence		DALYs		QCI	
	Rate per (100,000), 2017	Percent change (%), 1990 to 2017	Rate per (100,000), 2017	Percent change (%), 1990 to 2017	Rate per (100,000), 2017	Percent change (%), 1990 to 2017	QCI (%), 2017	Percent change (%), 1990 to 2017
Neonatal disorders	853.6 (808.4 to 913.4)	-2.2 (-3.6 to 42.8)	2641.0 (2338.2 to 2977.3)	28.7 (23.0 to 4.6)	6542.7 (6220.5 to 6858.0)	-44.6 (-48.4 to -23.9)	91.2	17.2
Respiratory infection and tuberculosis	280971.0 (234225.1 to 332179.3)	-2.5 (-3.6 to -41.0)	21273.1 (17970.3 to 25334.6)	-11.2 (-13.6 to 1.5)	3340.5 (3100.2 to 3594.6)	-67.7 (-70.4 to -1.4)	88.9	17.2
Enteric infections	99984.3 (87841.0 to 114137.4)	-0.6 (-4.2 to -71.8)	1513.4 (1347.3 to 1695.8)	-4.6 (-8.1 to -7.7)	2575.3 (2296.7 to 2879.4)	-66.1 (-71.0 to 3.7)	85.3	34.1
Dermatitis	2221.2 (1833.0 to 2653.5)	0.4 (-0.4 to -3.1)	5286.0 (4861.0 to 5734.8)	3.1 (1.8 to 1.0)	225.8 (123.9 to 372.9)	3.8 (2.4 to 32.5)	53.1	9.0
Asthma	1059.4 (816.3 to 1325.3)	-7.2 (-14.9 to 54.2)	4117.2 (3224.8 to 5101.3)	-5.2 (-13.2 to 4.2)	210.1 (145.2 to 295.4)	-39.5 (-50.3 to -48.6)	90.8	12.1
Leukemia	4.3 (3.8 to 4.9)	-18.1 (-41.0 to -12.0)	34.2 (29.0 to 38.6)	12.4 (-23.0 to 45.3)	151.6 (134.4 to 167.4)	-41.3 (-56.6 to 12.7)	65.4	69.3
Road injuries	408.2 (313.2 to 535.5)	-1.5 (-9.1 to -42.1)	264.0 (233.2 to 296.3)	-5.6 (-7.8 to -4.6)	572.2 (542.0 to 601.1)	-49.1 (-53.3 to -63.7)	91.4	8.4
Mental disorders	2842.8 (2543.6 to 3240.4)	3.8 (2.6 to 7.6)	8915.4 (8049.8 to 9843.7)	-0.4 (-1.5 to -3.3)	901.8 (638.6 to 1223.7)	3.2 (2.0 to 0)	21.8	10.6
Foreign body	293.7 (228.8 to 370.4)	7.2 (5.1 to 1.5)	106.6 (80.8 to 137.3)	2.8 (-0.1 to 45.1)	155.4 (142.8 to 169.0)	-55.7 (-60.0 to -2.1)	93.4	9.4
Idiopathic epilepsy	47.7 (35.3 to 63.4)	12.3 (-4.1 to 12.1)	377.4 (287.2 to 490.1)	16.7 (-0.5 to 36.0)	222.2 (167.9 to 288.5)	-22.5 (-35.0 to 6.0)	81.1	31.3
Diabetes mellitus	65.7 (49.2 to 83.0)	32.8 (26.1 to -24.4)	340.0 (261.2 to 426.4)	36.2 (30.4 to 47.4)	37.3 (29.6 to 47.2)	-3.1 (-12.4 to -53.0)	79.7	21.1

Data in parenthesis are 95% Uncertainty Interval (UI)

### Respiratory infection and tuberculosis

Global deaths and DALY regarding these causes decreased in <20y population between 1990–2017 (Table 1). This cause takes 12.2% of DALY in <20y. (Table 1, S43 Fig in S2 File). Overall global estimated QCI score is 88.9. QCI scores in different SDI quintiles are 99.3, 94.9, 92.4, 84.5 and 83.1 for high, high-middle, middle, low-middle, and low SDI regions respectively. Nigeria has the lowest score and Spain has the highest score (S3 Table in S1 File, S32 Fig in S2 File). Global QCI scores represent a growing trend over a period of 28-year. Although, the growth of the index in low-SDI quintiles was significant, a notable gradient remains between low and high SDI countries (Fig 3). In 2017, all SDI quintiles had an equal condition of care between two sexes, as GDR score in all ages was just near one (Fig 4).

### Enteric infections

Global deaths and DALY due to diarrheal, typhoid and paratyphoid diseases notably decreased in <20y population between 1990–2017 (Table 1, S44 Fig in S2 File). It takes 9% of DALY in <20y. The estimated global QCI score is 85.3. Estimation of this index in different SDI quintiles is 97.2, 96.3, 93.1, 82.8 and 78.3 for high, high-middle, middle, low-middle, and low SDI regions. The lowest score goes to Central African Republic and the highest goes to Austria (S4 Table in S1 File, S33 Fig in S2 File). Global QCI score shows an increasing pattern between 1990 and 2017 with a significant change in low SDI quantiles. Overall, changes in QCI scores over the time represent a convergent shape in different SDI quantiles (Fig 3). In 2017, across all ages, GDR scores were just near one in all SDI quintiles, representing an equal condition of care between two sexes (Fig 4).

### Mental disorders

DALYs due to mental disorders -including (ordered by the highest burden to the lowest) conduct disorder, anxiety disorders, depressive disorders, idiopathic developmental intellectual disability, autism spectrum disorders, bipolar disorders, eating disorders, attention-deficit/hyperactivity disorders (ADHD) and schizophrenia- in <20y population, increased between 1990–2017 (Table 1, S45 Fig in S2 File). Mental health takes 3% of DALY in <20y. Overall estimation of global QCI score is 21.8. Estimation of this score in different SDI quintiles is 37.1, 26.2, 22.0, 18.7 and 15.4 for high, high-middle, middle, low-middle, and low SDI regions. Estonia has the highest score, and lowest score belongs to India (S5 Table in S1 File, S34 Fig in S2 File). Time trend of QCI score shows a steady pattern, representing a significant gradient between developed and developing countries (Fig 3). Across all age groups (excluding neonatal and 1-4y) in different SDI quantiles, unequal distribution of quality care is evident, favoring better cares in females (Fig 4).

### Road injuries

Global death and DALY due to road injuries in <20y population decreased between 1990–2017 (Table 1, S46 Fig in S2 File). 2% of DALY in <20y population belongs to road injury. Estimated global QCI score is 91.4. The calculated score in different SDI quintiles is 97.6, 94.6, 91.6, SDI 87.8 and 86.2 for high, high-middle, middle, low-middle, and low SDI regions. The highest score is for New Zealand while the lowest score belongs to Haiti (S6 Table in S1 File, S35 Fig in S2 File). Global QCI score represent a rising trend between 1990 and 2017 (Fig 3). Overall changes in QCI scores over the time show a convergent pattern in different SDI quantiles. All SDI quintiles have experienced equality in quality of care between sexes, as GDR score in all ages have narrowed just near one (Fig 4).

### Dermatitis

DALYs due to dermatitis in <20y population increased between 1990–2017 (Table 1, S47 Fig in S2 File). 0.8% of DALY in under 20y held by dermatitis. Overall estimation of global QCI score is 53.1. This score in different SDI quintiles was calculated 50.0, 42.2, 50.5, 55.2 and 60.8 for high, high-middle, middle, low-middle, and low SDI regions. The highest score belongs to Peru and the lowest score goes to North Korea (S7 Table in S1 File, S36 Fig in S2 File). Global QCI score shows a steady trend (Fig 3). In 2017, GDR scores across all ages were just near one, representing an equality in condition of care between sexes (Fig 4).

### Idiopathic epilepsy

The rate of DALY in population of >20y decreased between 1990–2017 (Table 1, S48 Fig in S2 File). It takes 0.8% of DALY in <20y. Global estimated QCI score is 81.0. Calculation of QCI score in different SDI quintiles is 97.5, 90.8, 89.0, 74.5 and 72.7 for high, high-middle, middle, low-middle, and low SDI regions. The highest score goes to Singapore, while Mali has the lowest score (S8 Table in S1 File, S37 Fig in S2 File). Time trend of QCI score between 1990–2017 shows a growing pattern and low SDI quintiles represent higher gradient growth over time (Fig 3). Overall, care quality between sexes shows equality in distribution. In low SDI countries gender disparities was evident in 10-14y population pro girls and in 1-4y population pro boys, which has been eliminated until 2017 (Fig 4).

### Asthma

Deaths and DALY due to asthma in <20y population reduced during a period of 28 years (Table 1, S49 Fig in S2 File). Asthma takes 0.8% of DALY in <20y. The global estimated QCI score is 90.8. Calculated QCI scores in different SDI quintiles are 95.5, 94.0, 91.7, 89.7 and 86.7 for high, high-middle, middle, low-middle, and low SDI regions. Netherland has the highest score, while Central African Republic has the lowest score (S9 Table in S1 File, S38 Fig in S2 File). Global QCI trend shows a slow rising pattern during a 28-year period and low-SDI quintiles have experienced a significant growth (Fig 3). In 2017, across all ages, GDR scores were just near one in all SDI quantiles (Fig 4).

### Foreign body

Global deaths and DALY in <20y due to foreign body decreased between 1990–2017 (Table 1, S50 Fig in S2 File). 0.6% of DALY in <20y belongs to foreign body. There is no difference in QCI across countries with different SDI. Laos has the lowest score, and the highest score belongs to Japan (S10 Table in S1 File, S39 Fig in S2 File). Globally, the QCI score represented a rising trend between 1990–2017 and a convergent pattern is seen in gradient between different SDI quantiles (Fig 3). Equality in distribution of qualified care is evident between sexes, as GDR scores across all ages are just near one in all SDI quantiles (Fig 4).

### Leukemia

Global deaths and DALY due to leukemia in <20y population decreased between 1990–2017 (Table 1, S51 Fig in S2 File). Leukemia takes 0.6% of DALY in <20y. Overall global QCI score is estimated 65.4. Calculation of this index in different SDI quintiles is 88.3, 90.0, 71.0, 30.0 and 24.7 for high, high-middle, middle, low-middle, and low SDI regions. UK has the highest score, while the lowest score belongs to Swaziland (S11 Table in S1 File, S40 Fig in S2 File). Global QCI score has represented a growing trend over time. While this score raised in high, high-middle and middle-SDI regions in a period of 28-years, low and low-middle SDI area showed a steady pattern during this time, which led to a gradient between wealthy and underdeveloped countries (Fig 3). In 2017, all SDI quintiles had similar care quality in both sexes, as GDR score in all ages was just near one (Fig 4).

### Diabetes mellitus

Rate of DALY due to DM type1 (T1DM) and type2 (T2DM) in <20y population, decreased during 28 years (Table 1, S52 Fig in S2 File). DM takes 0.1% of DALY in >20y. Estimation of global QCI score is 79.7. QCI scores in different SDI quintiles are 92.8, 87.2, 83.3, 74.2 and 74.0 for high, high-middle, middle, low-middle, and low SDI regions. Tajikistan has the lowest score, and the highest score belongs to Cyprus (S12 Table in S1 File, S41 Fig in S2 File). Trend of global QCI score during the study period was growing. Changes in high-middle quintiles is more significant. Although the score in all SDI quantiles has an increasing pattern, disparities remain between high and low SDI countries (Fig 3). Equal distribution of quality care is evident among sexes, as GDR scores across all ages are just near one in all SDI quantiles in 2017 (Fig 4).

## Discussion

### Principal findings

Significant Improvement of the global QCI score in children and adolescents for respiratory infection, enteric infection, leukemia, foreign body aspiration, asthma, epilepsy, DM, dermatitis, road injury, and neonatal disorders during a period of 28 years is a marker of better pediatric health care. Mental health shows a very slow increasing pattern and disparities favoring females is evident. Evidences in 1990 shows a better condition of care pro girls in epileptic disorders for 1-4y (S27 Fig in S2 File) and 10-14y age groups (S29 Fig in S2 File) in low-SDI countries which moved toward equity over time. Other causes represent an equal distribution in qualified care in all age group and all SDI quantiles (S1-30 Fig in S2 File). We found out the care quality for important causes of death and morbidity in children and adolescents is notably higher in developed areas. The higher SDI quintiles have higher QCI scores and lower SDI quintiles have lower QCI scores.

### Interpretation

Globally, trend of DALYs in children and adolescents shows a significant improvement from 1990 to 2017 [8]. The role of global health policies is inevitable in this progress. By 2015, the main focus of global health policies was on the reduction of mortality in children [2, 3], including improvement in vaccination coverage [19], which is vividly seen in a faster reduction of DALYs in fetal causes in comparison with nonfatal causes [8, 9], as we found that the trend of care quality for mental disorders was unsatisfying.

Mental disorders care is a challenging problem while sociopolitical and cultural/familial factors alter in different countries [20]. Guidelines were described to modify the help seeking behavior of affected families [21]. Understanding the mental health problems such as mood disorders, autism, absent seizures, mental retardation, and ADHD, may be difficult for some families especially in mild to moderate disorders; therefore, guided protocols for case finding may improve care quality for mental disorders [22].

The importance of adolescent’s health was globally missing for years and recently it came to the agenda [7]. The result of this neglect is evident in a decreased gradient of DALYs between the highest SDI quintiles and the lowest SDI quintiles in adolescent age group [8]. In this study, we found that the care quality for respiratory disease, and enteric infection are higher in younger childhood than adolescence. Adolescent age group are more parent-independent in comparison with younger children, so they have authority in their management while they are not intellectually and financially independent enough [23].

Global and local investments to developing comprehensive strategies over the years [24, 25] has led to valuable outcomes encompassing reduction of mortality rate due to asthma to nearly a quarter, upgrading the survival rate of leukemia, improvement in neonatal mortality and morbidity index, and near half decrease in mortality due to road injury. Although developing countries are in a shortage of qualified infrastructures, comprehensive programs were successful in improvement the condition even in these countries. Despite such improvements, the gap between infrastructures still made an inequality. Lack of financial and human resources to effectively allocate the care of patients remains a challenge in low-income countries [12]. Furthermore, universal policies and resources are still needed to strengthen transportation infrastructures and elimination of environmental risk factors.

Respiratory and enteric infection were globally the leading causes of death in children. The global policies and practical guideline following adoption of MDG in 2000 [2] have made remarkable changes in 2015 and 2017 [8]. The focus of MDG on reduction of mortality in under 5-year-olds led a better care quality in them rather than older children.

The rise in prevalence and incidence of DM could be the result of: 1) further detection of T1DM [26] and T2DM in screening programs and following a further awareness in health care providers [27] 2) extension of unhealthy life style in children and adolescents and raising pattern in prevalence of obesity in this age group, as a major risk factor for T2DM [27, 28]. Development of local programs for prevention, early detection and effective treatment, impacts on better control of the challenge.

DALYs due to mental disorders in children and adolescents has raised over time, therewith unsatisfying trend of improvement in quality of care. Implementation of mental health policies for children and adolescents is still a challenge in low setting areas, which could be due to low health literacy, political unwillingness, lack of a global agenda for mental health for children, stigma and shortage of resources [29]. Gender disparity in mental health quality of care is evident. This could arise from 1) higher prevalence of conduct disorders and ADHD in boys [30] 2) stigma in support seeking in males which results in late interventions [31]. Local policies and programs, alongside a sustainable support from international and non-governmental organizations, contribute to enhancing mental health coverage and capacity [32, 33].

Dermatitis has different pattern which could be due to the following explanations. 1) Limited data, few countries reported national registry data 2) Dermatitis is more prevalent in developed countries (5378.77(4737.68, 6086.50) cases per 100,000 in low SDI versus 8648.52(8209.39, 9145.36) cases in high SDIs). The higher prevalence of dermatitis in high SDI regions is related to the theory of microbiome, which indicates that a higher exposure to microbes could have a protective impact on atopic dermatitis [34]. 3) In North America, incidence and prevalence of dermatitis is high which could be related to a large population of non-white ethnic groups, who have higher prevalence rather than white ethnic group [35]. On the other hand, presentations of dermatitis in black skin cases are diagnosed with delay, leading to a more severe manifestation [35]. Thus, access to oriented healthcare staff and affordable treatment is a challenge for declining the burden of this cause [15].

The diagnosis of foreign body aspiration at the right time and emergent medical interventions, along with preventive educations are comprehensive strategies to decline accidents leading to death [36]. The unexpected pattern in gradient of QCI among different SDI quantiles could be due to the missing data, since only 34 countries reported the relevant data.

### Strengths and limitations

In this study we report the situation of care quality for important causes of mortality and morbidity in children and adolescents by sex and age, which is a novel report among the burden of disease studies. While IHME reported HAQI (as a quality index) only by 32 different causes, and they did not report sex or age differences [11].

There are several measures estimated and extracted from different models in GBD studies. Disparities could be found in some measures due to the following reasons: 1) as a result of poor capacity of that model in estimating specific conditions like rare events, 2) it could represent a real disparity in health outcomes, for example an odd prevalence of a disease in a country due to a poor health coverage for that specific disease. At first, we chose 30 causes, but according to the challenges in availability and validity of data, we have limited the study to 11 causes. Unfortunately, data from many countries is missing in section GHDx in IHME website which disrupts global resolutions. Based on computational challenges, we have not calculated uncertainty for QCI score.

## Conclusion

Global quality of care index which was introduced in this study revealed a universal growth from 1990 to 2017 in major causes of death and morbidity in children and adolescents. Except for mental health which represented a gender disparity in condition of care pro females, other causes showed equal distribution of qualified care for both sexes. We demonstrated that care quality is an associate of the level of country’s development. Hence, despite the recent achievements in the management of major causes of mortality and morbidity in children and adolescents, farther investments are required to allocate the resources more effectively and to overcome inequities.

## Supporting information

S1 File QCI validation results and QCI trend values by globe, SDI regions, and countries. (ZIP)

S2 File QCI and GDR by globe, SDI regions, and countries and percent changes in DALYs rate among countries. (PDF)
