# Supplementary material for: Global, regional, and national burden and quality of care index in children and adolescents: A systematic analysis for the global burden of disease study 1990–2017
Source: PLoS One. 2022 Apr 26;17(4):e0267596. doi: 10.1371/journal.pone.0267596 (PMC9041858; doi:10.1371/journal.pone.0267596)
Supplement: S1 File — (ZIP) [file pone.0267596.s001.zip › eTable 1- Quality of care index (QCI) validity analysis.docx]

| Cause | Pearson correlation coefficient | |
| --- | --- | --- |
|  | HAQI | Cause-specific HAQI |
| Respiratory infections and TB | 0.7654 |  |
| Enteric infections | 0.6717 |  |
| Neonatal disorders | 0.8098 | 0.7652 |
| Leukemia | 0.7835 | 0.8046 |
| Asthma | 0.6946 |  |
| Epilepsy | 0.7456 | 0.6349 |
| Mental disorders | 0.7235 |  |
| Diabetes mellitus | 0.5671 | 0.4375 |
| Dermatitis | 0.1247 |  |
| Low back pain | 0.7997 |  |
| Road injury | 0.6924 |  |
| Foreign body | 0.4577 |  |
