# Supplementary material for: Global, regional, and national burden and quality of care index in children and adolescents: A systematic analysis for the global burden of disease study 1990–2017
Source: PLoS One. 2022 Apr 26;17(4):e0267596. doi: 10.1371/journal.pone.0267596 (PMC9041858; doi:10.1371/journal.pone.0267596)
Supplement: S2 File — (PDF) [file pone.0267596.s002.pdf]

Supplementary Figure 1

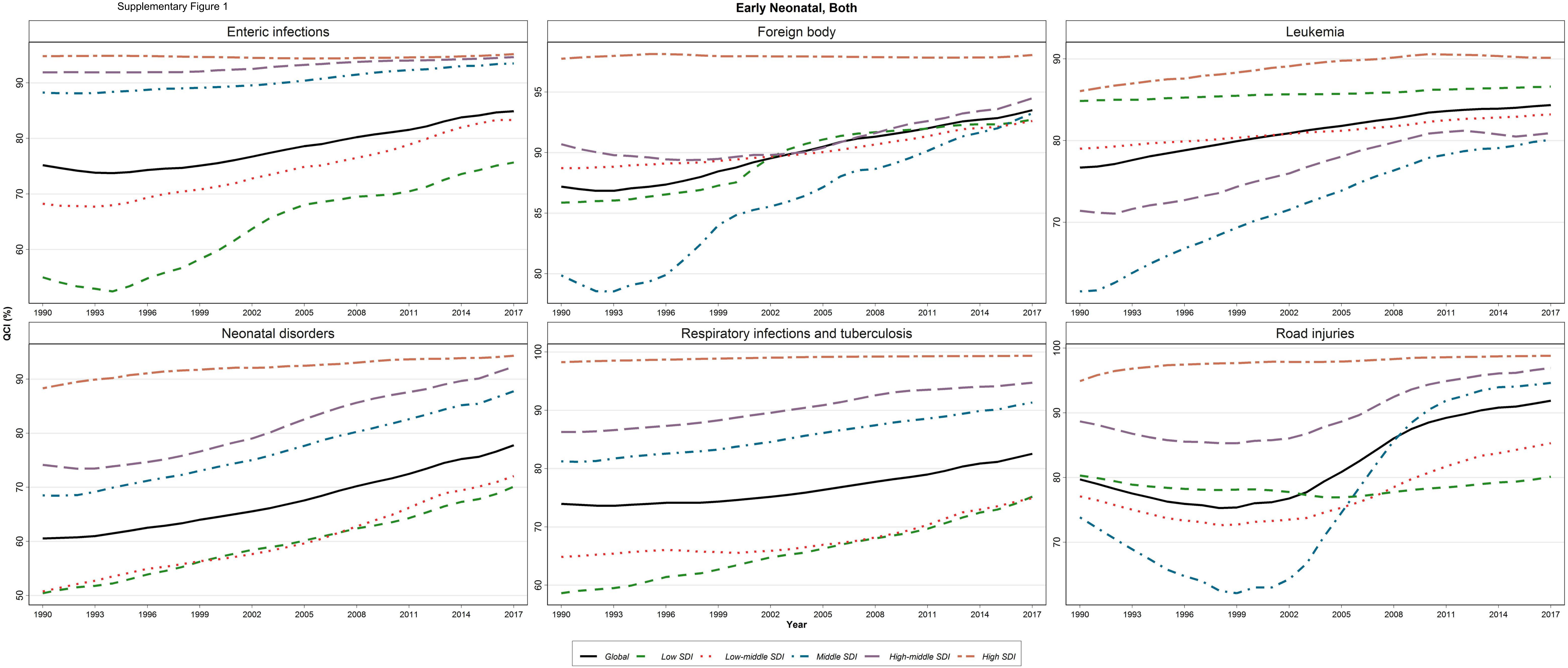

Supplementary Figure 2

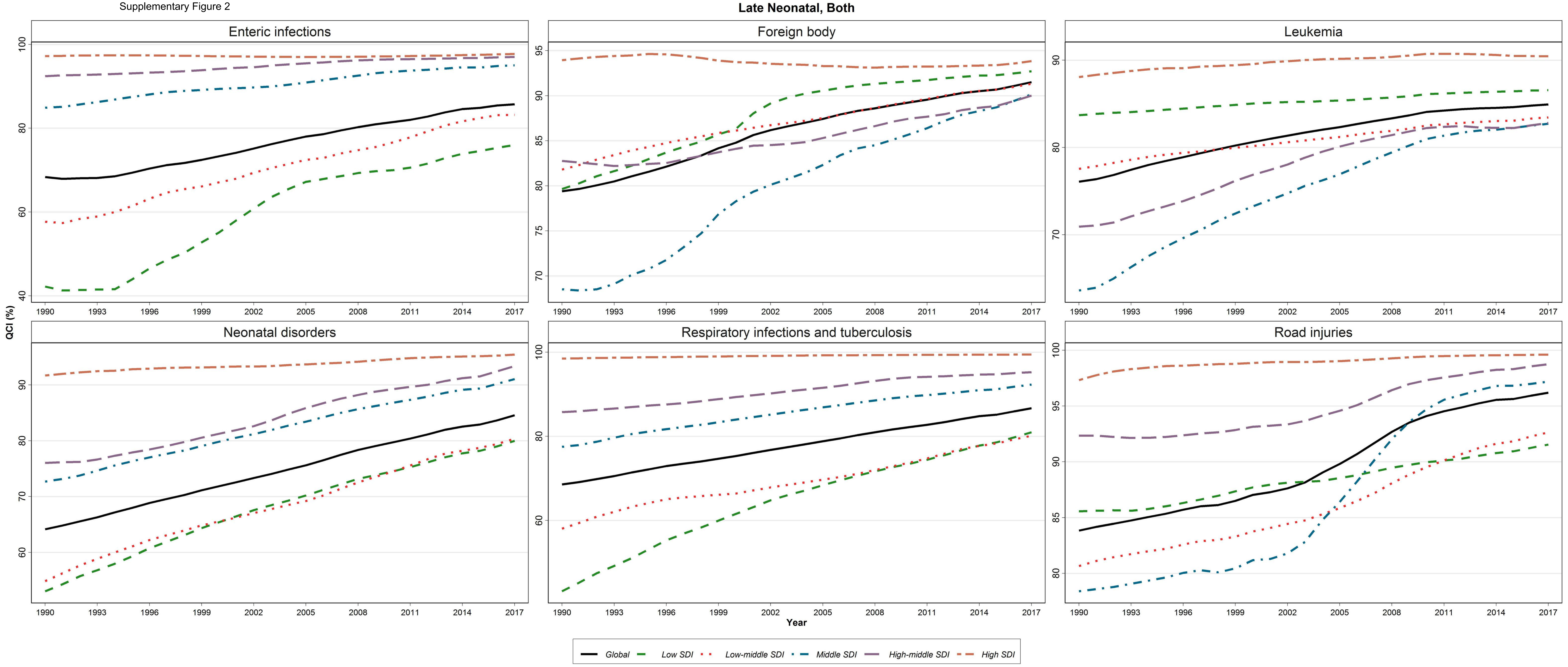

Supplementary Figure 3

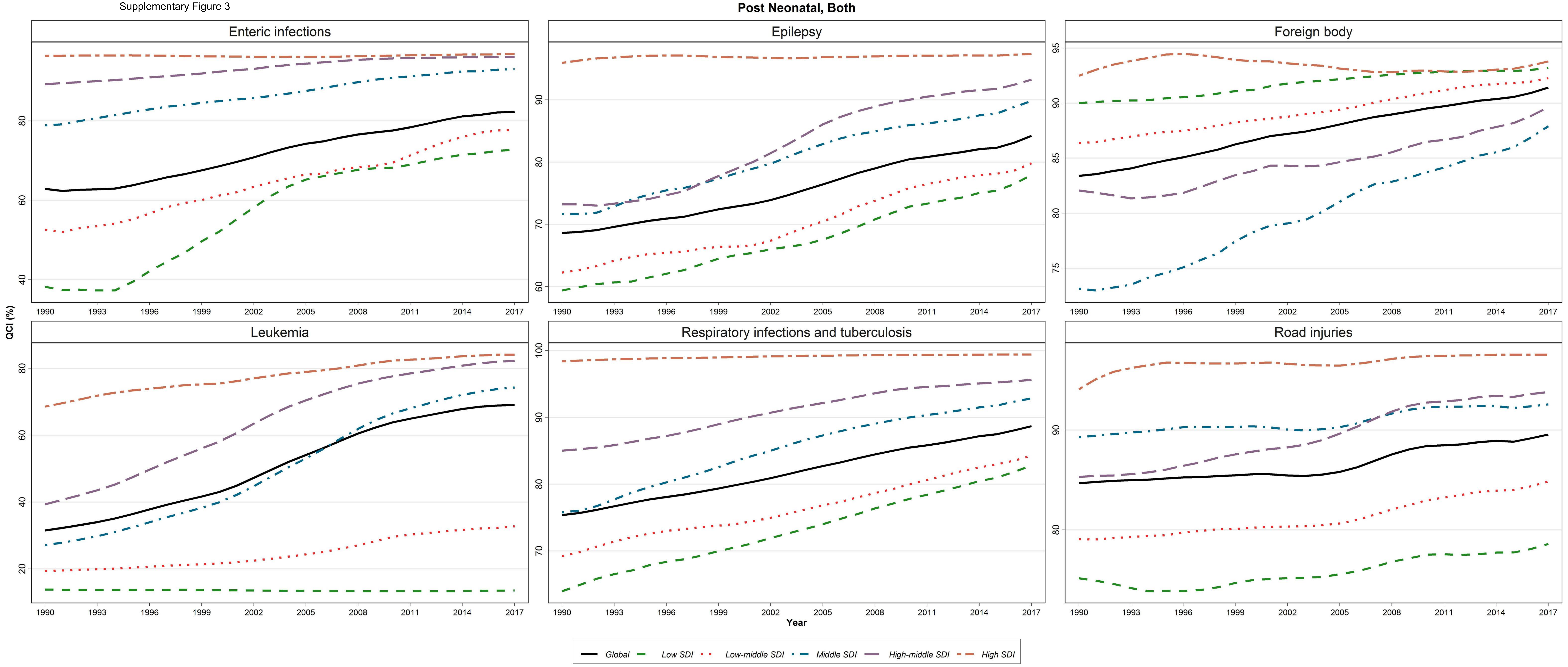

Supplementary Figure 4

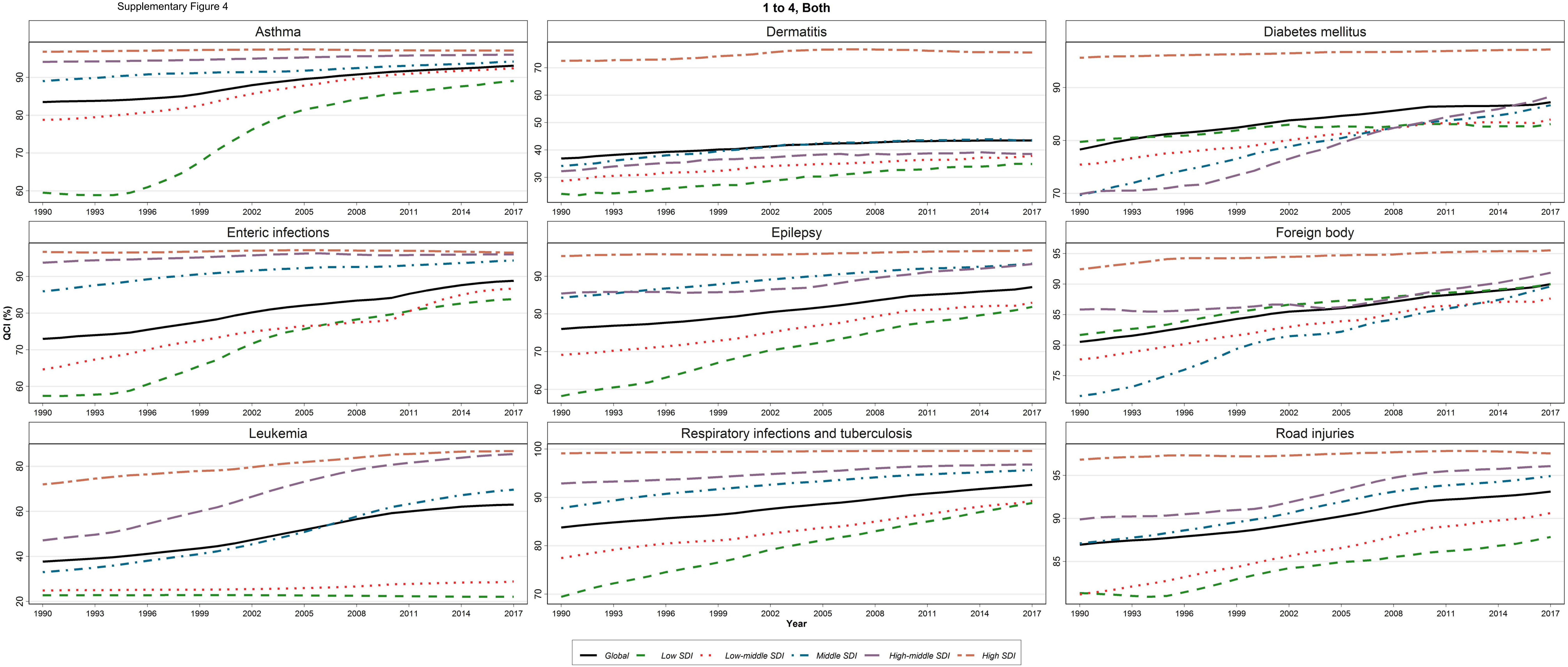

Supplementary Figure 5

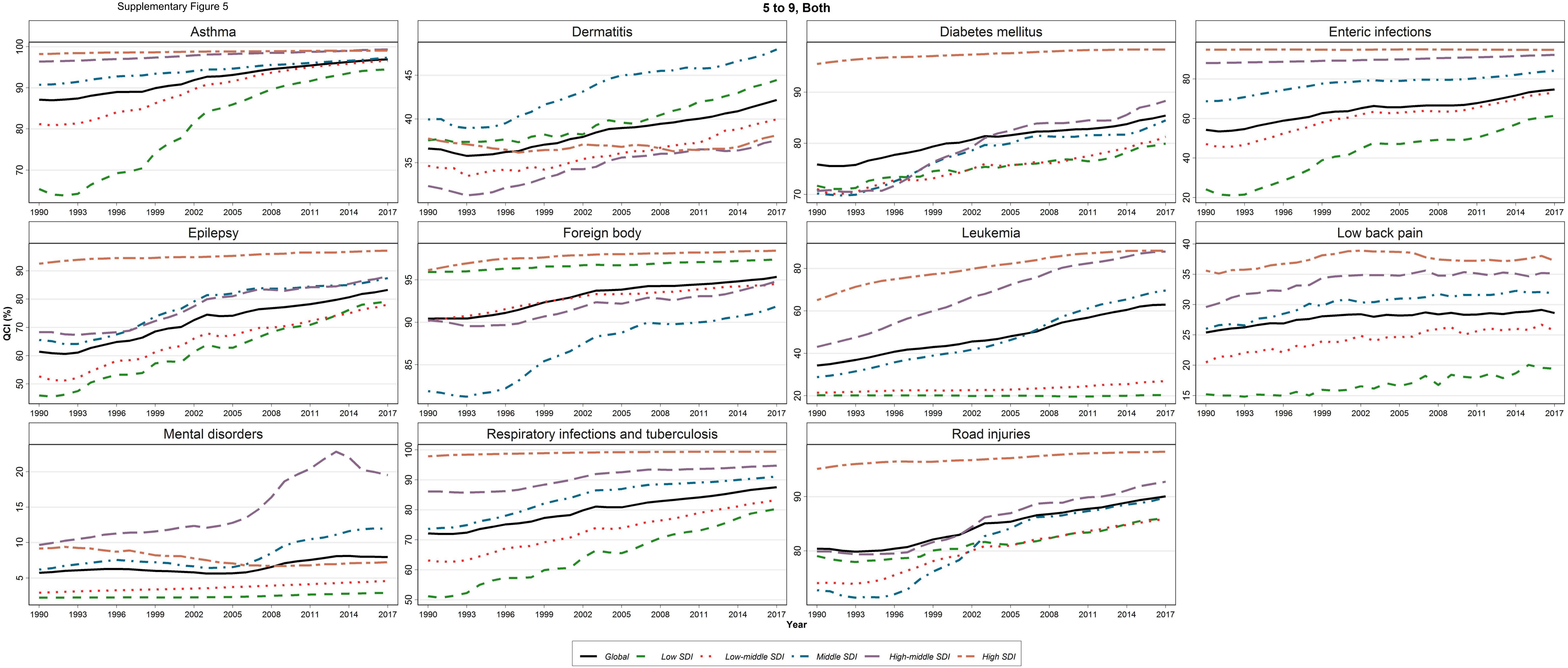

Supplementary Figure 6

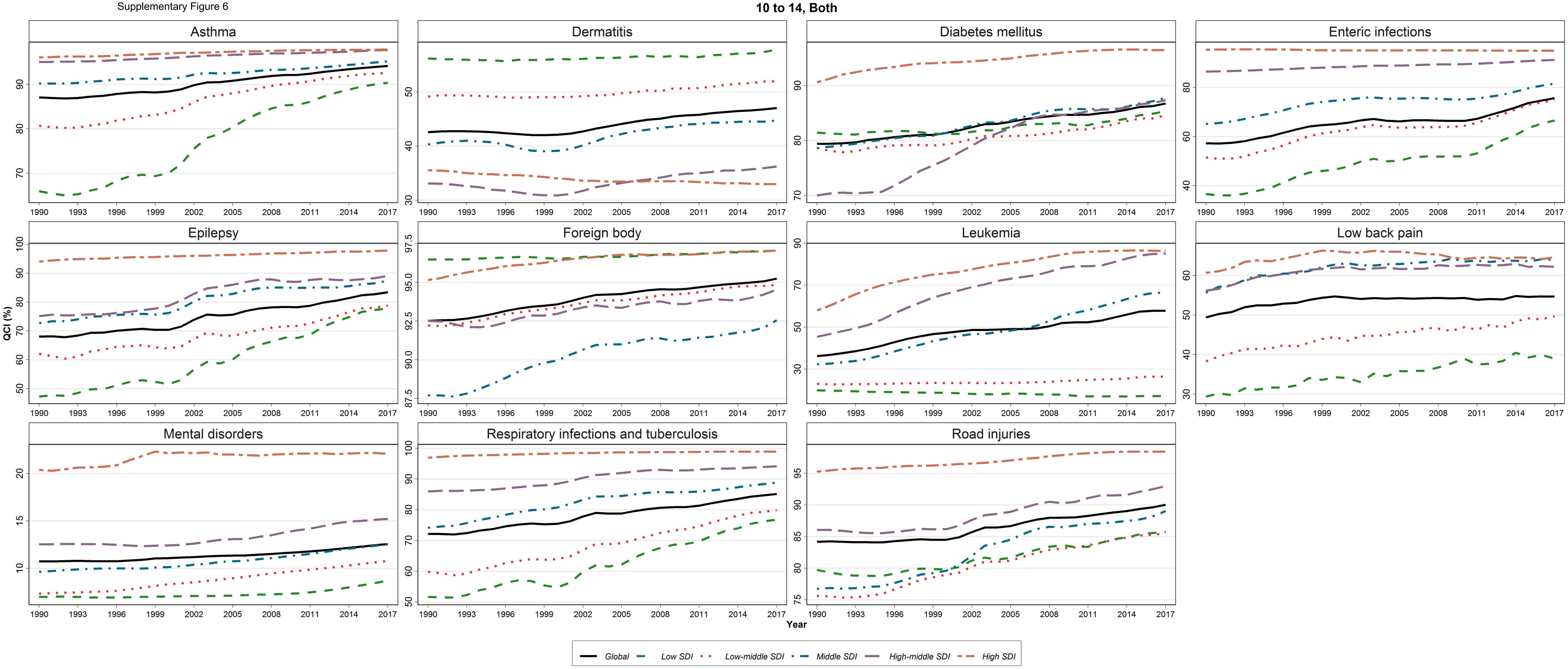

Supplementary Figure 7

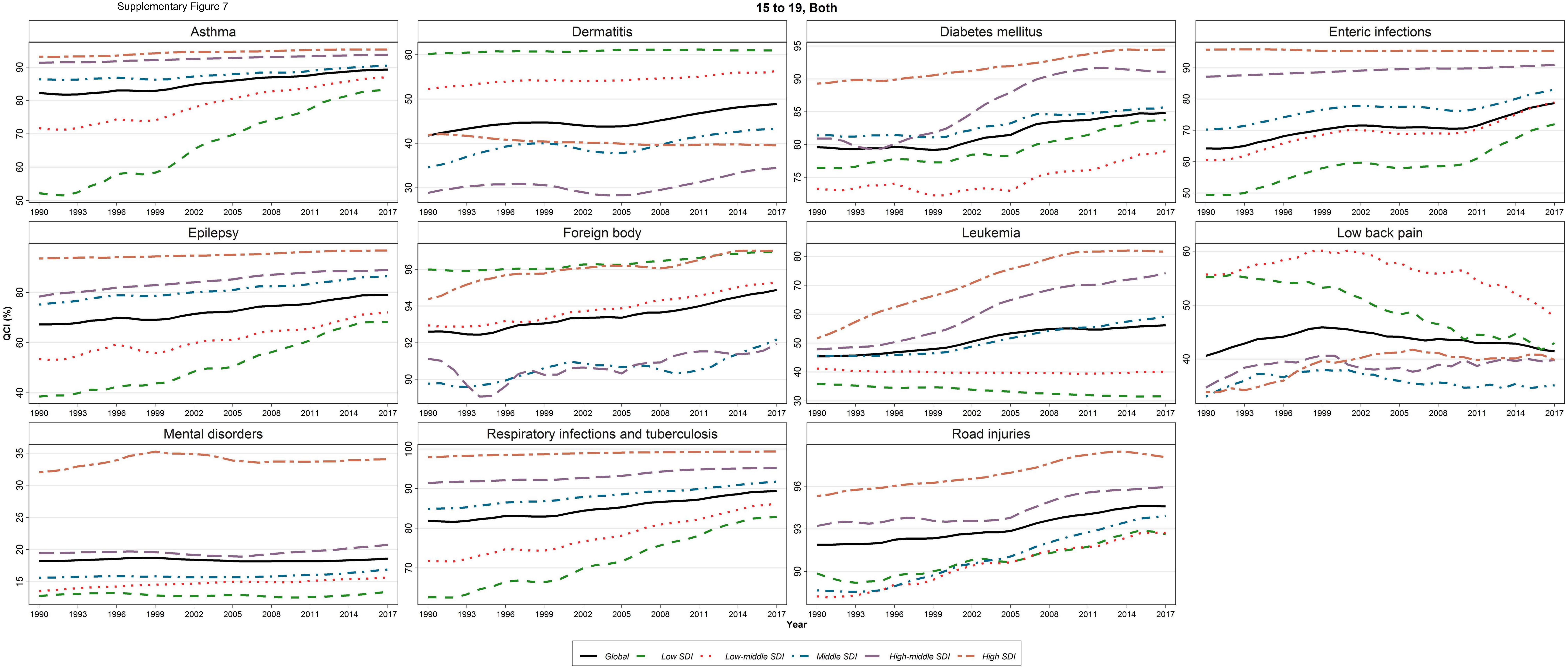

Supplementary Figure 8

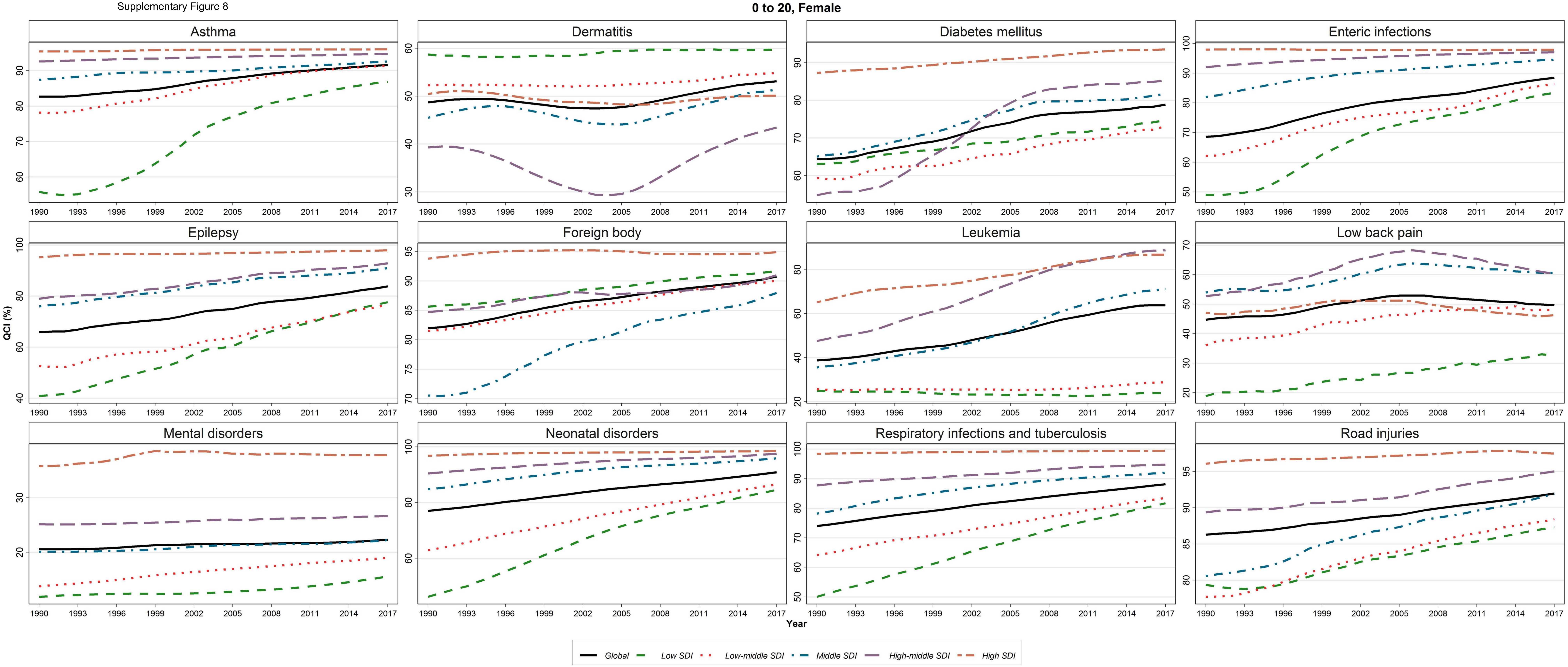

Supplementary Figure 9

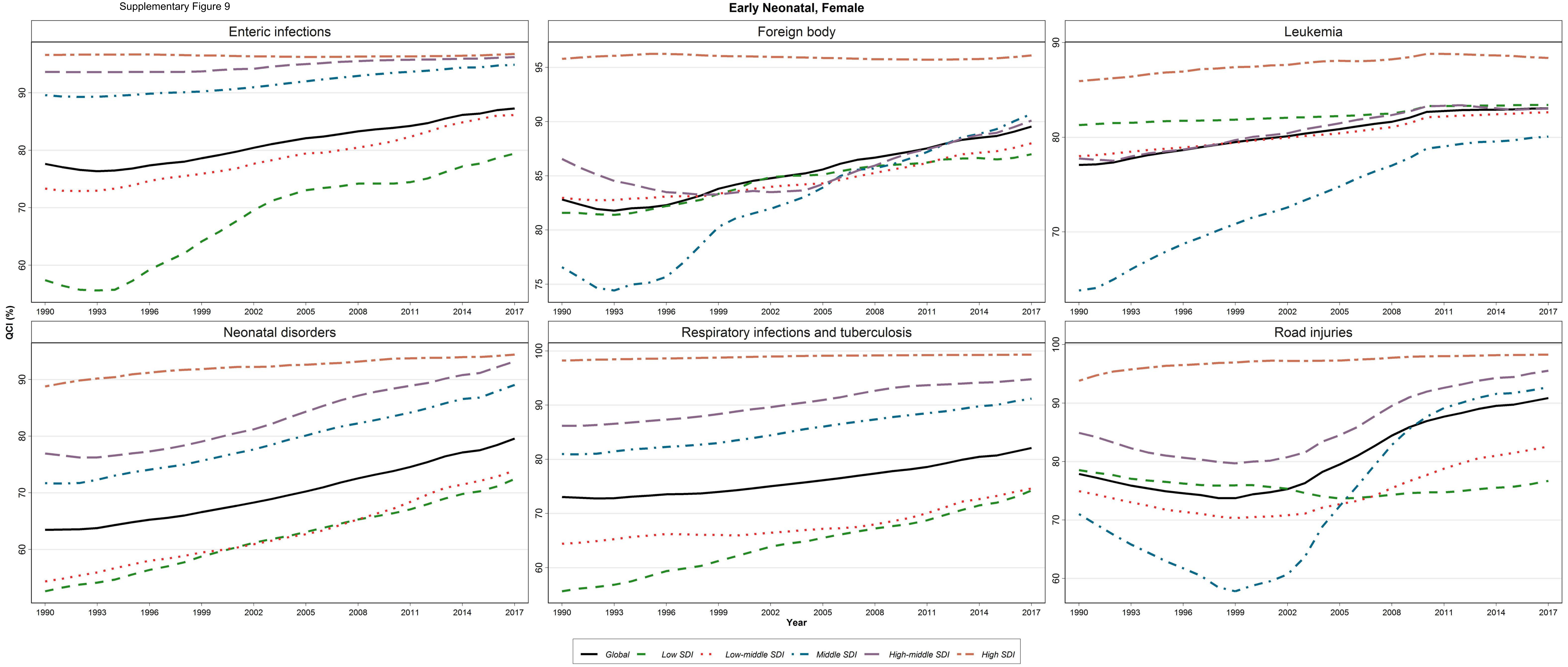

Supplementary Figure 10

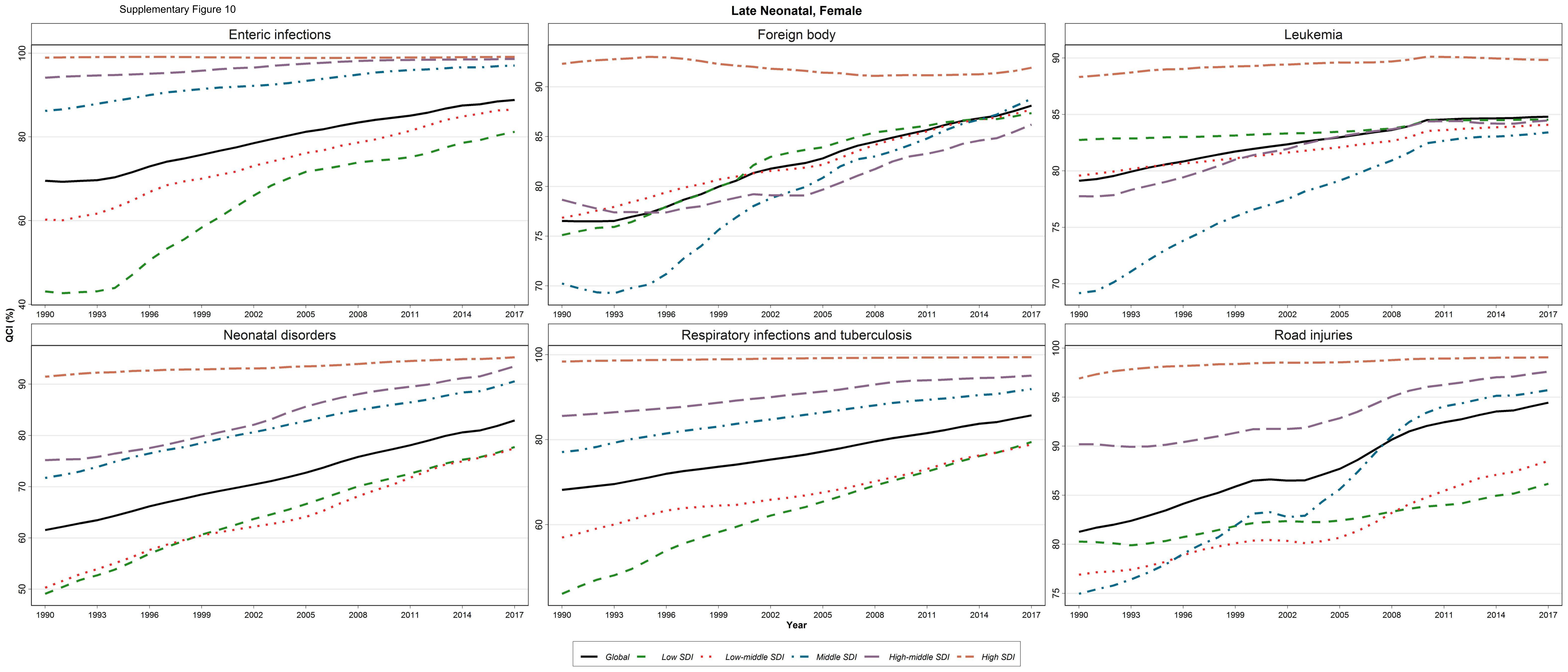

Supplementary Figure 11

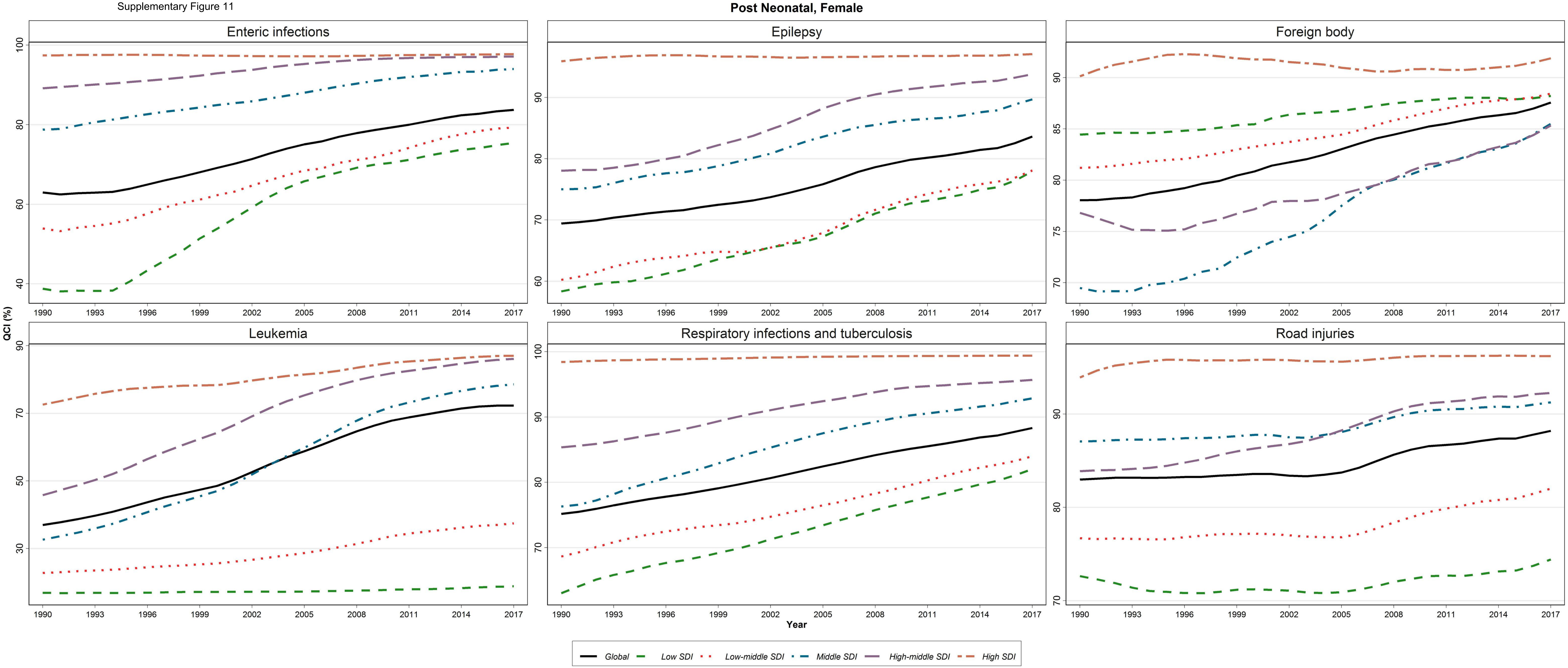

Supplementary Figure 12

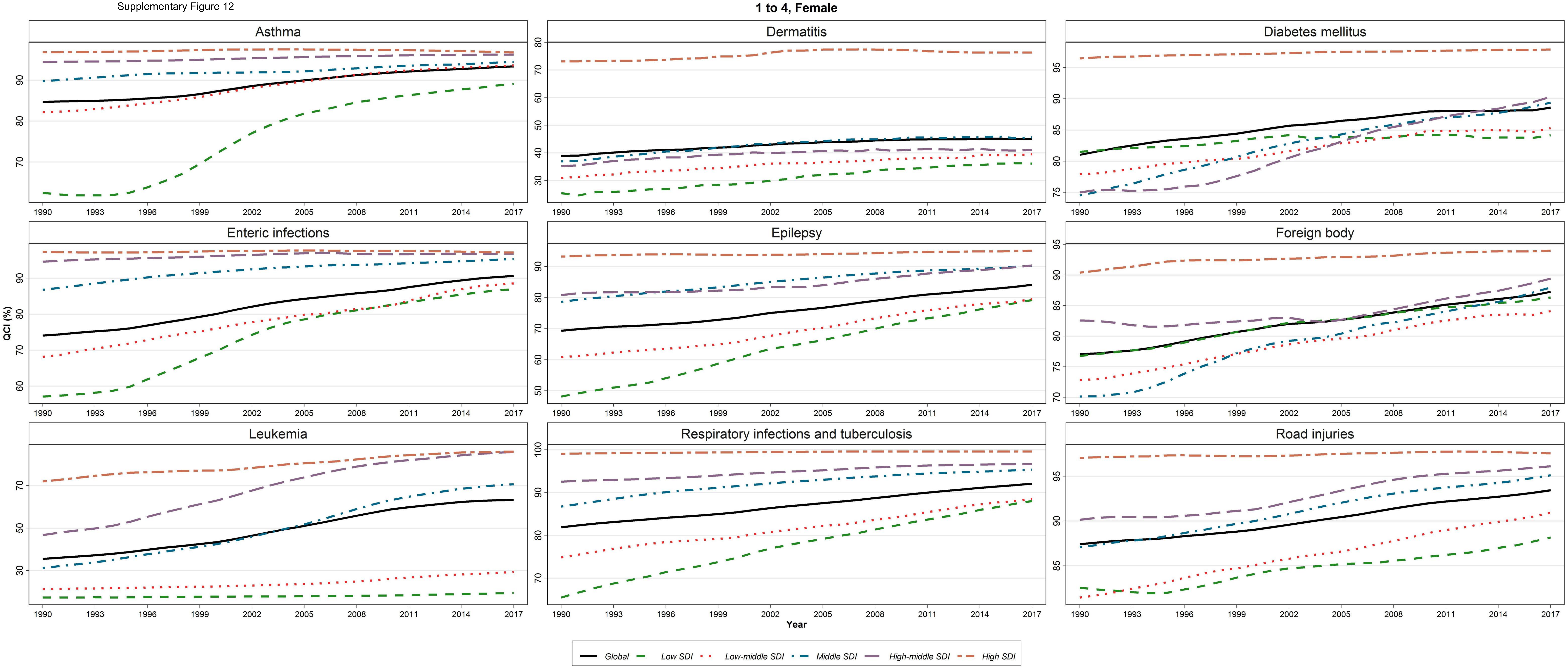

Supplementary Figure 13

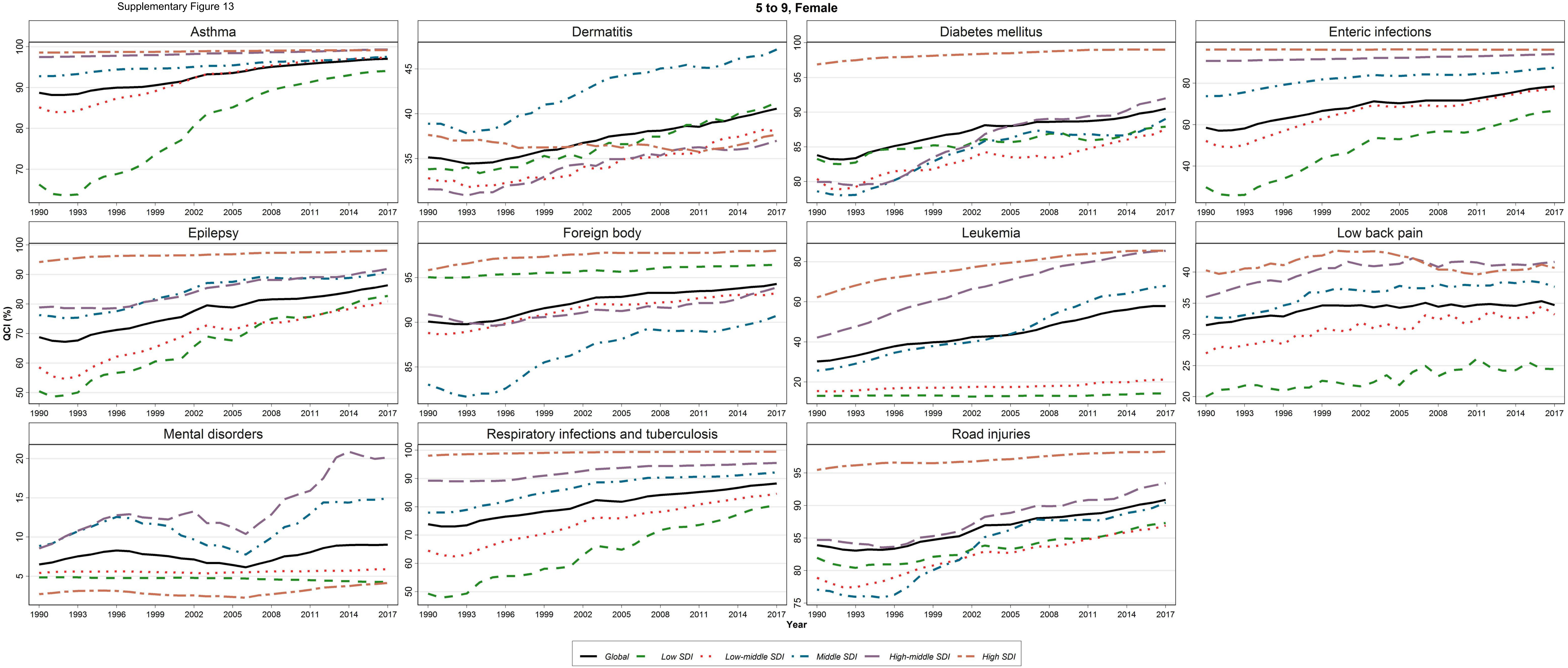

Supplementary Figure 14

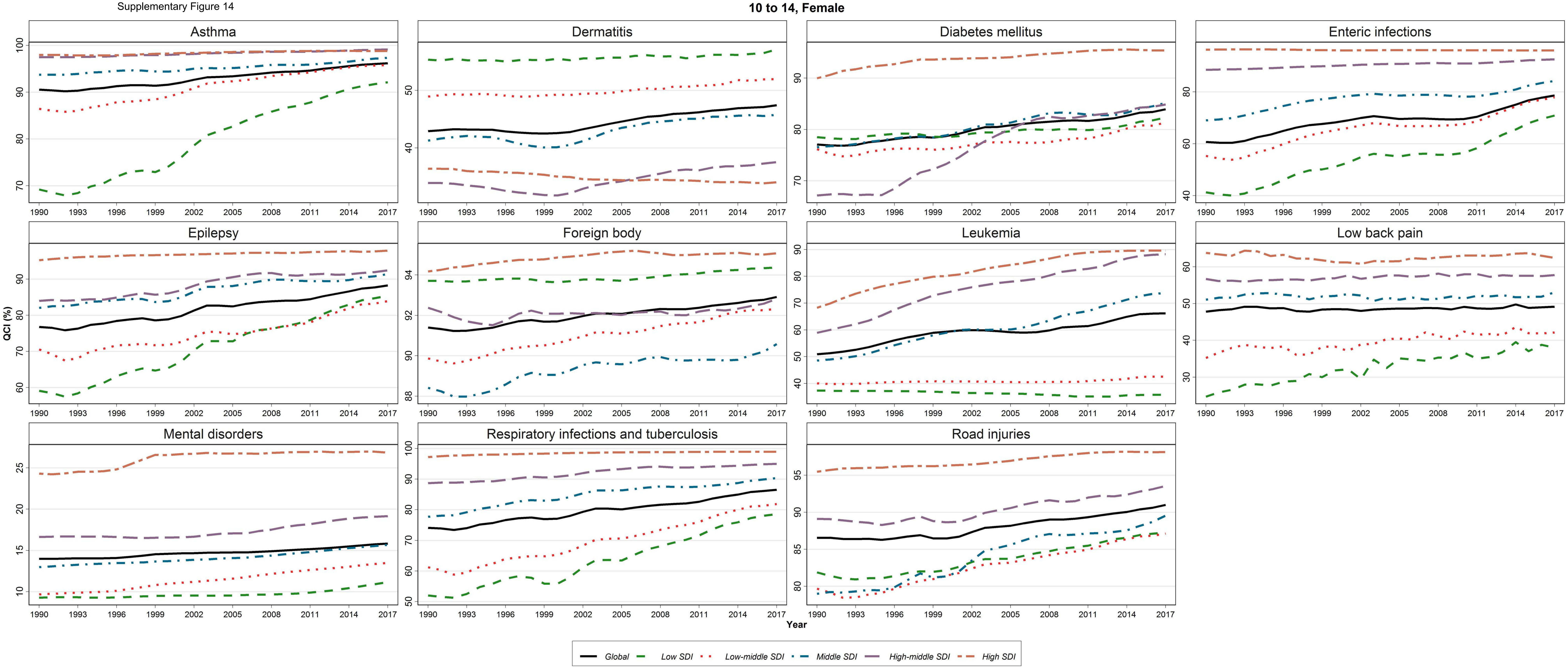

Supplementary Figure 15

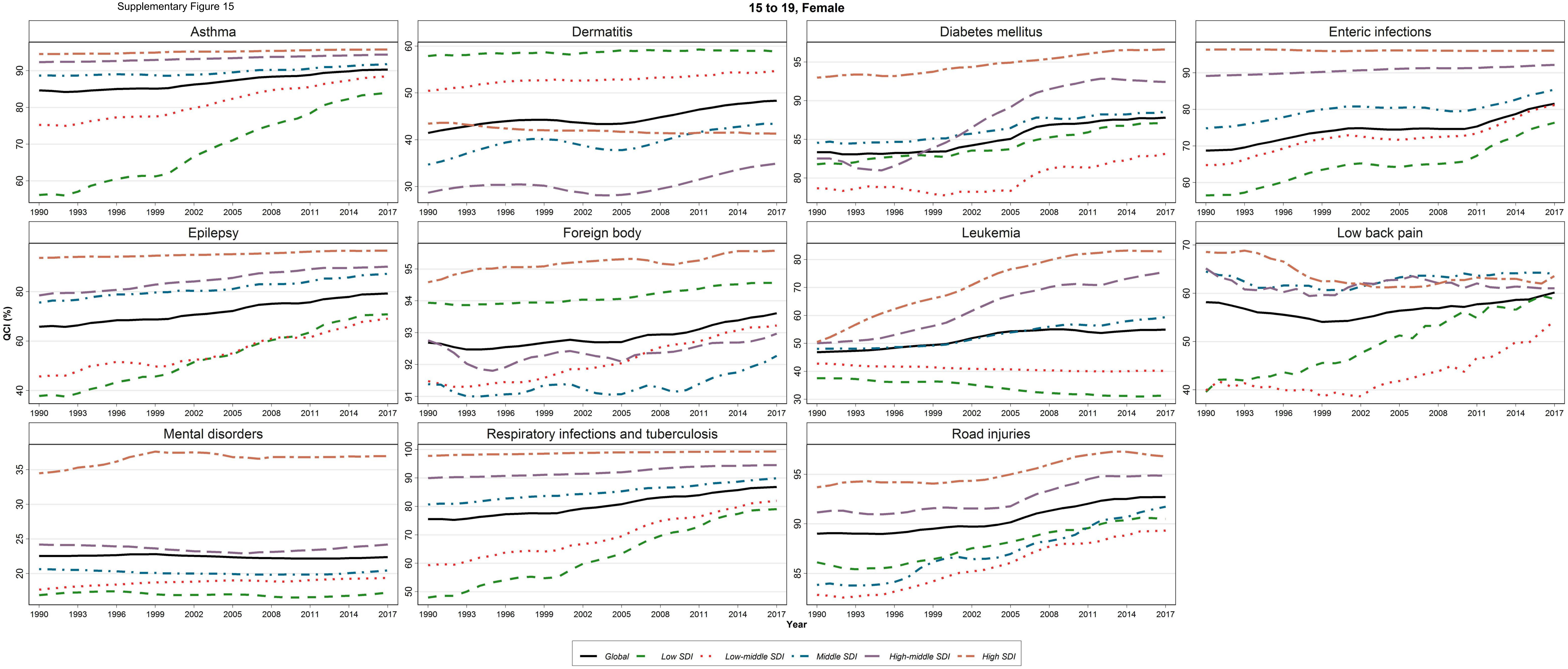

Supplementary Figure 16

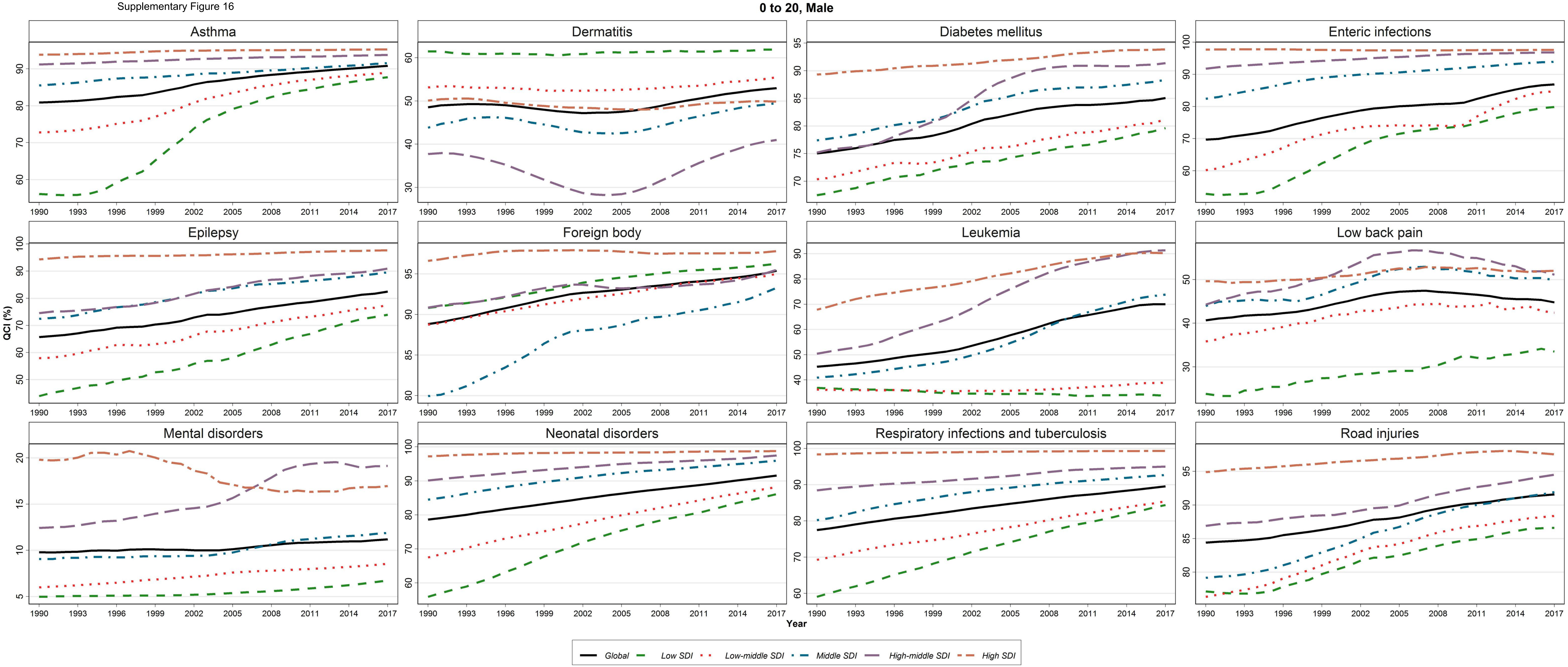

Supplementary Figure 17

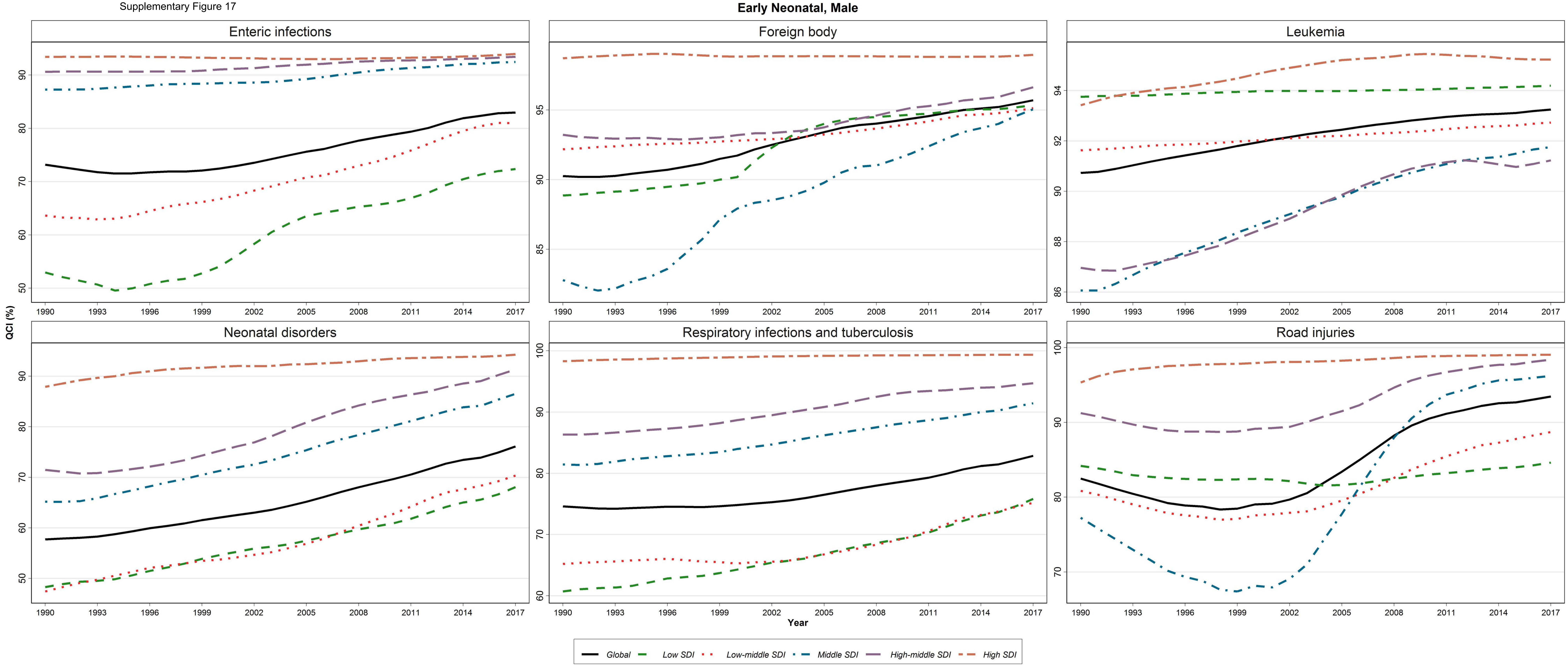

Supplementary Figure 18

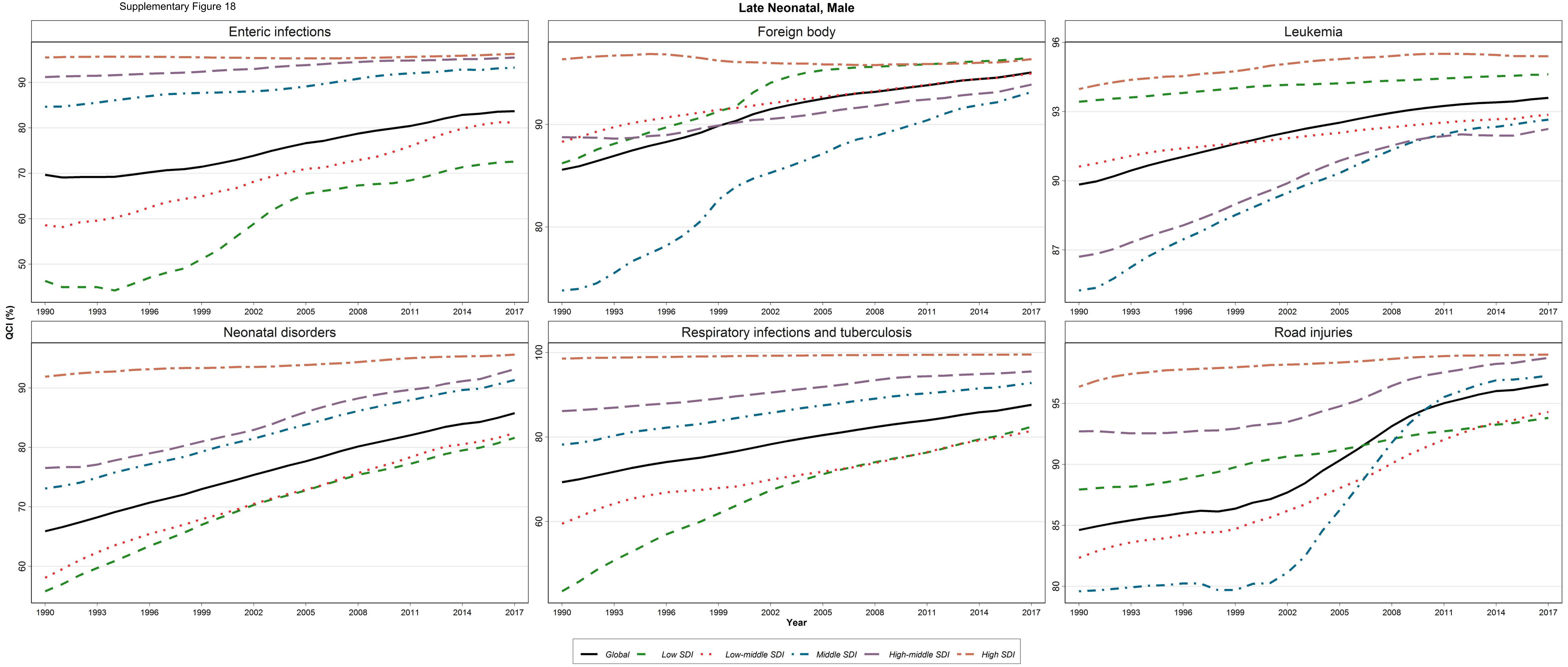

Supplementary Figure 19

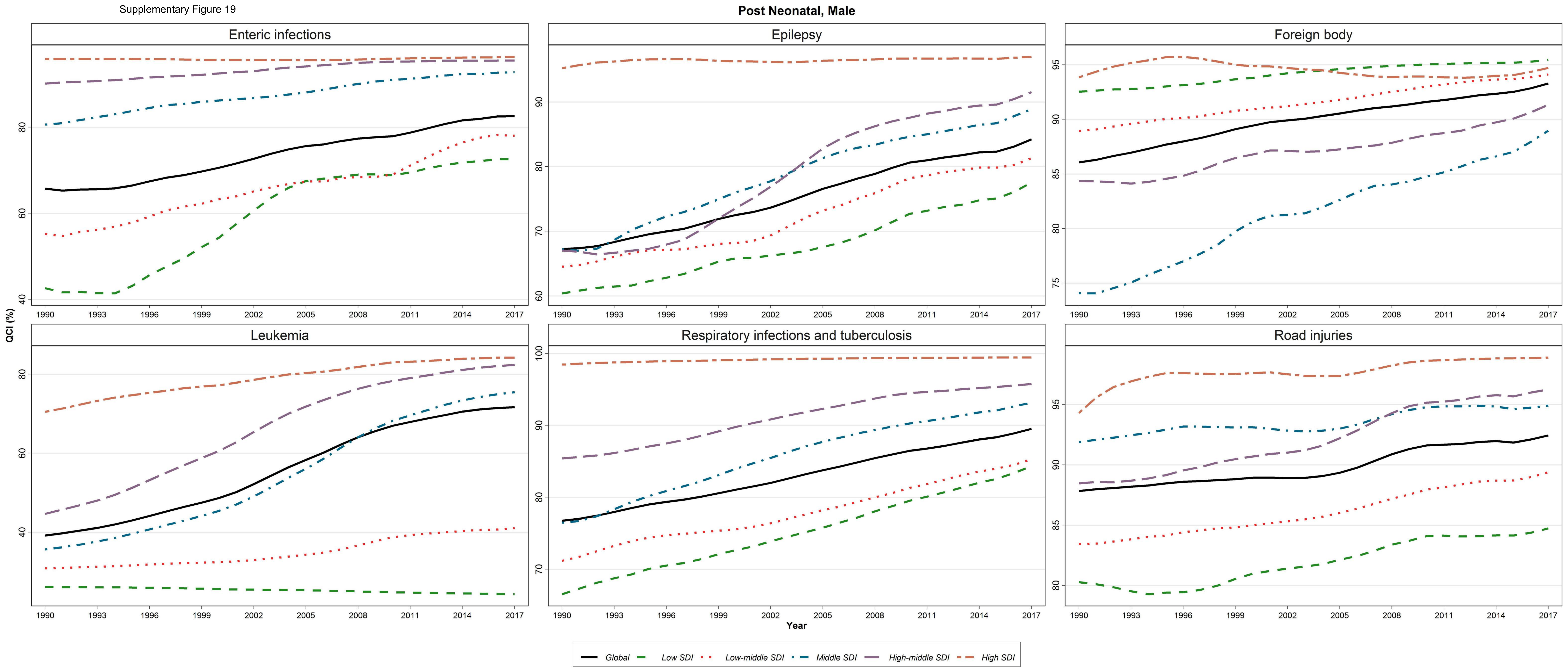

Supplementary Figure 20

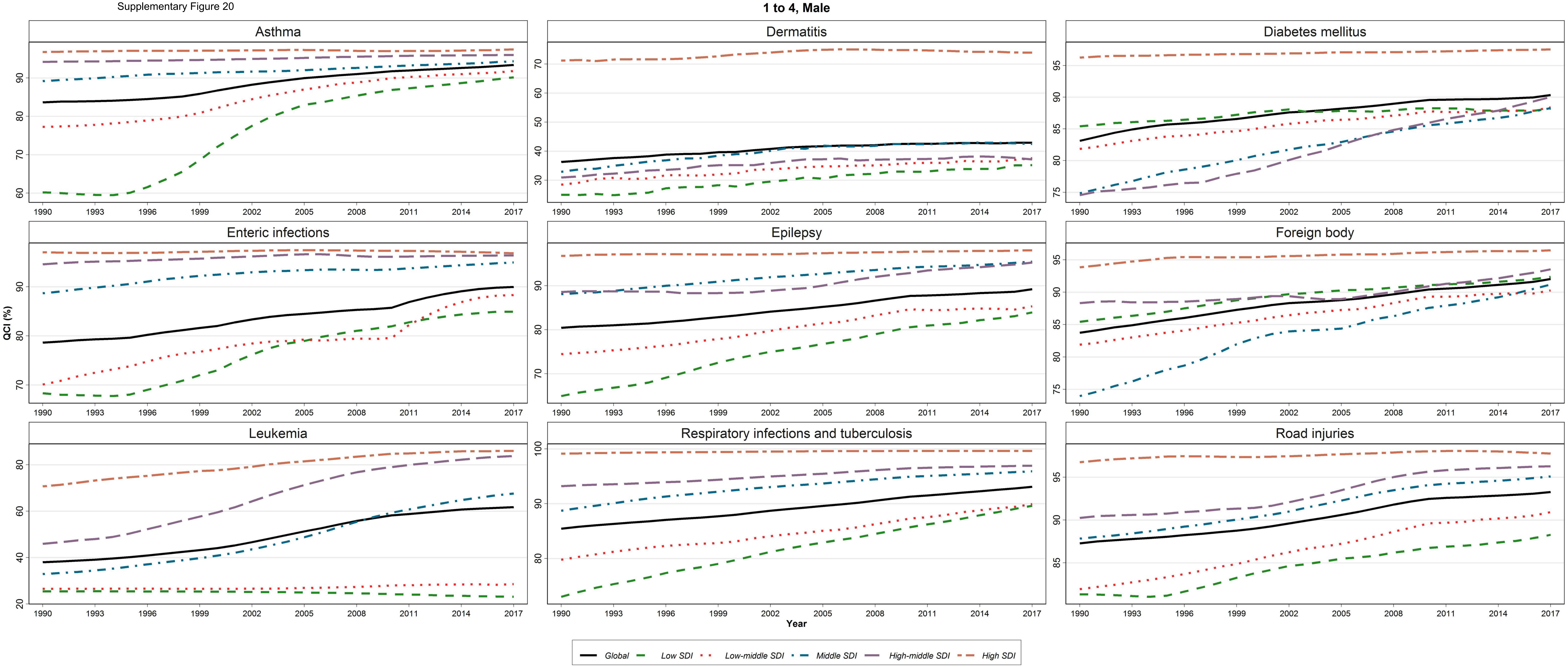

Supplementary Figure 21

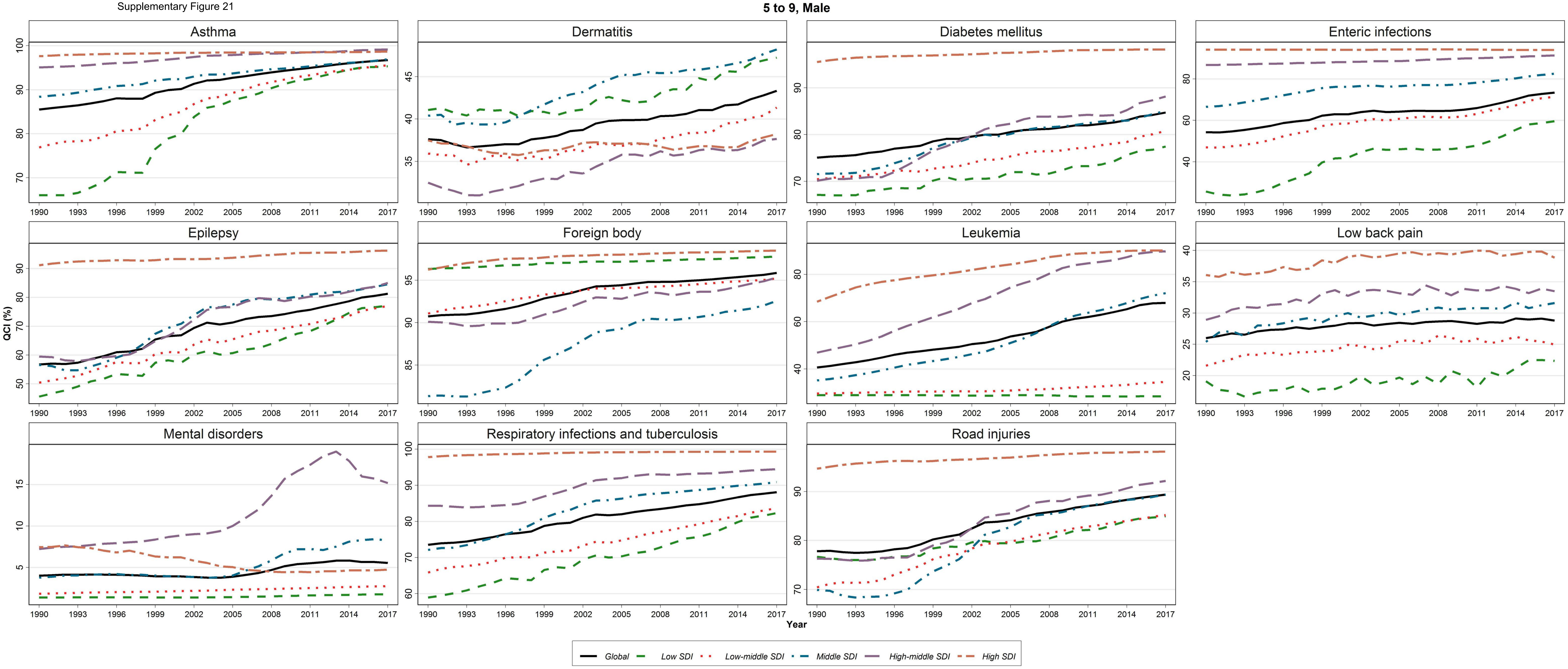

Supplementary Figure 22

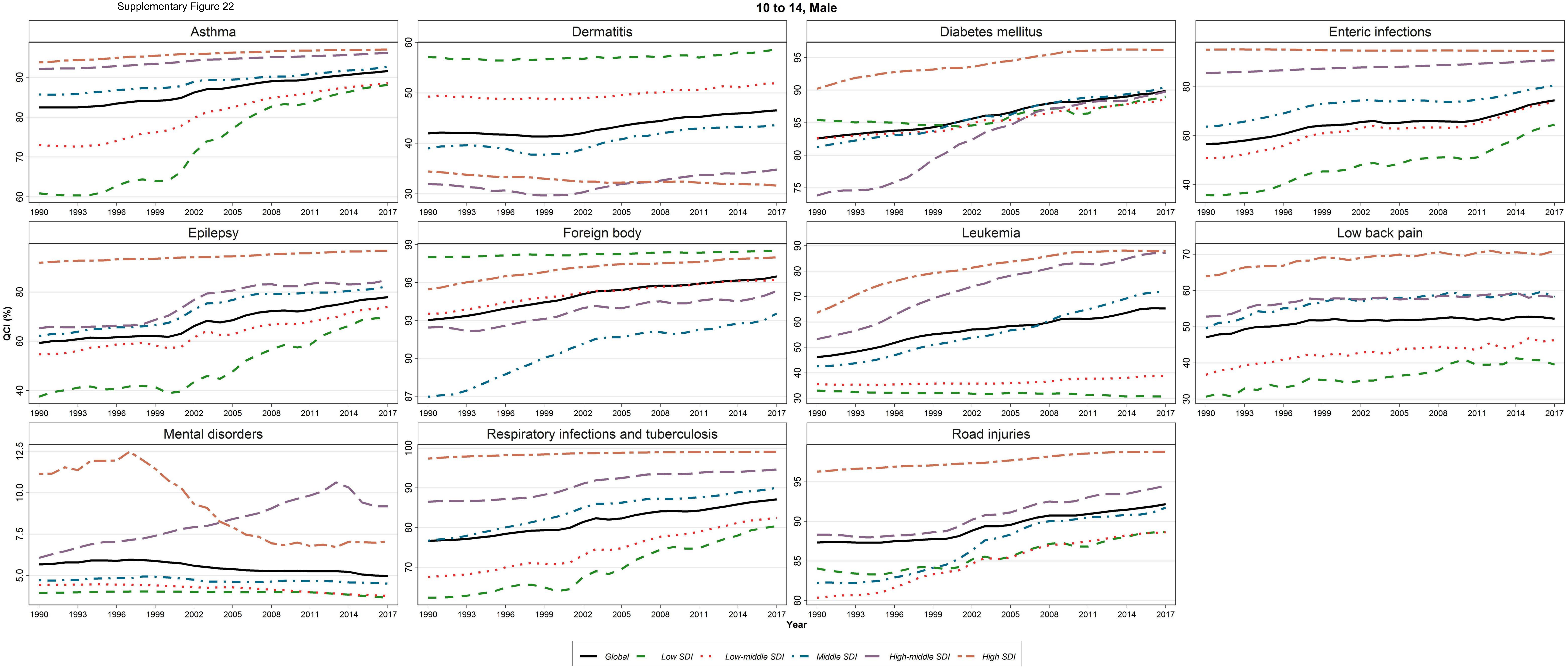

Supplementary Figure 23

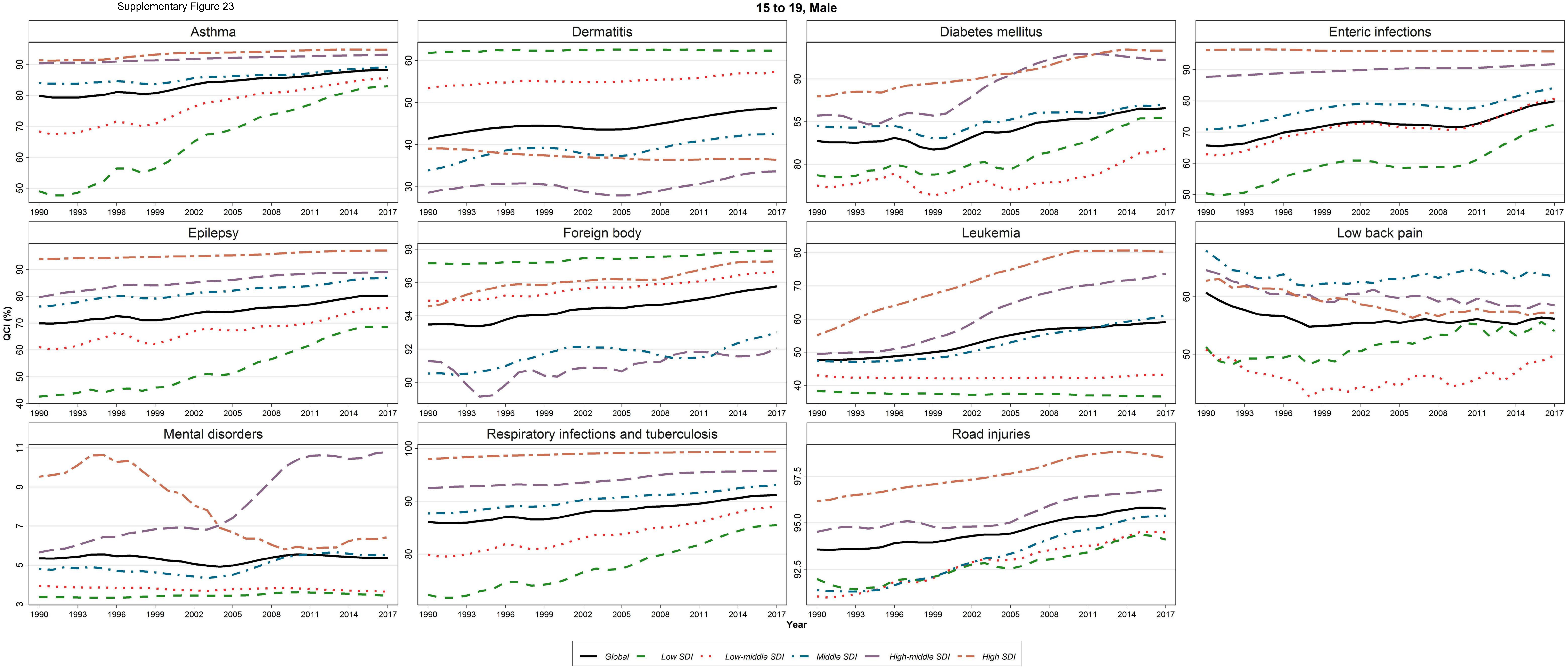

Supplementary Figure 24

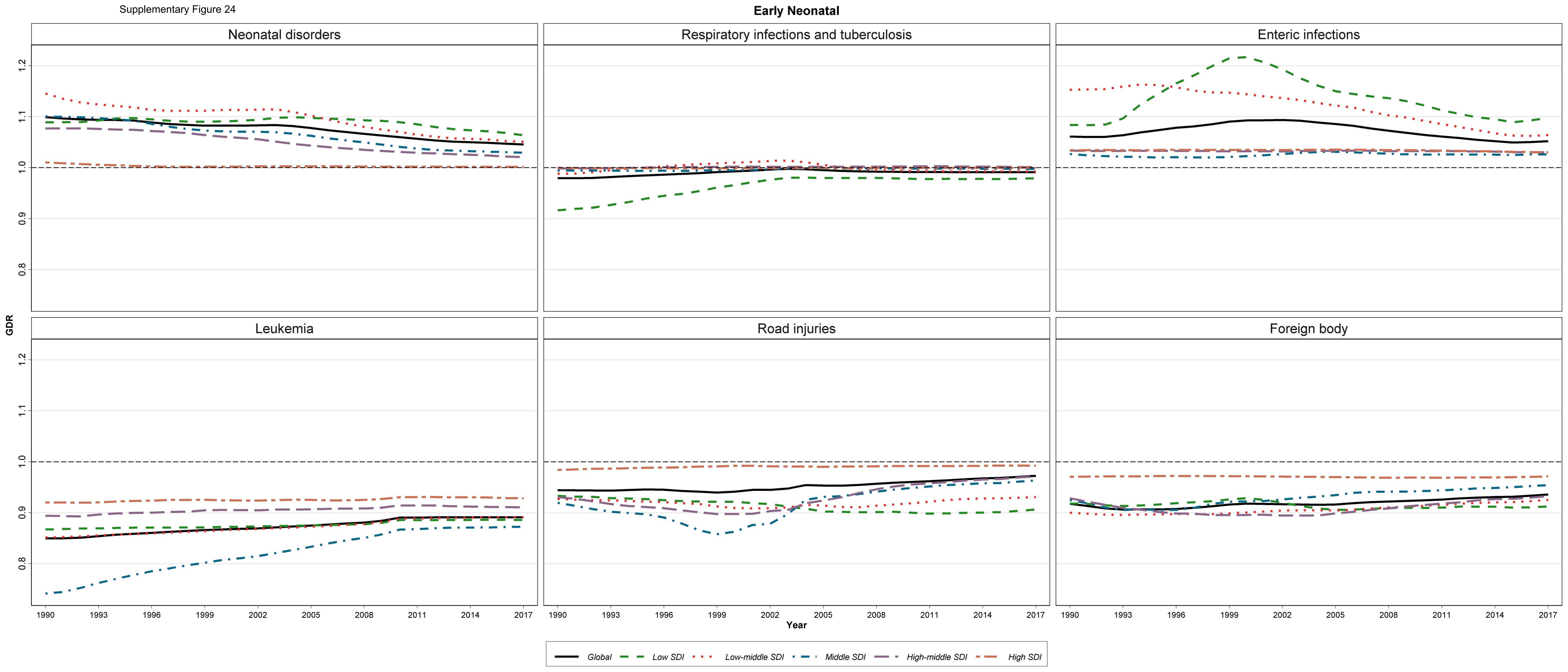

Supplementary Figure 25

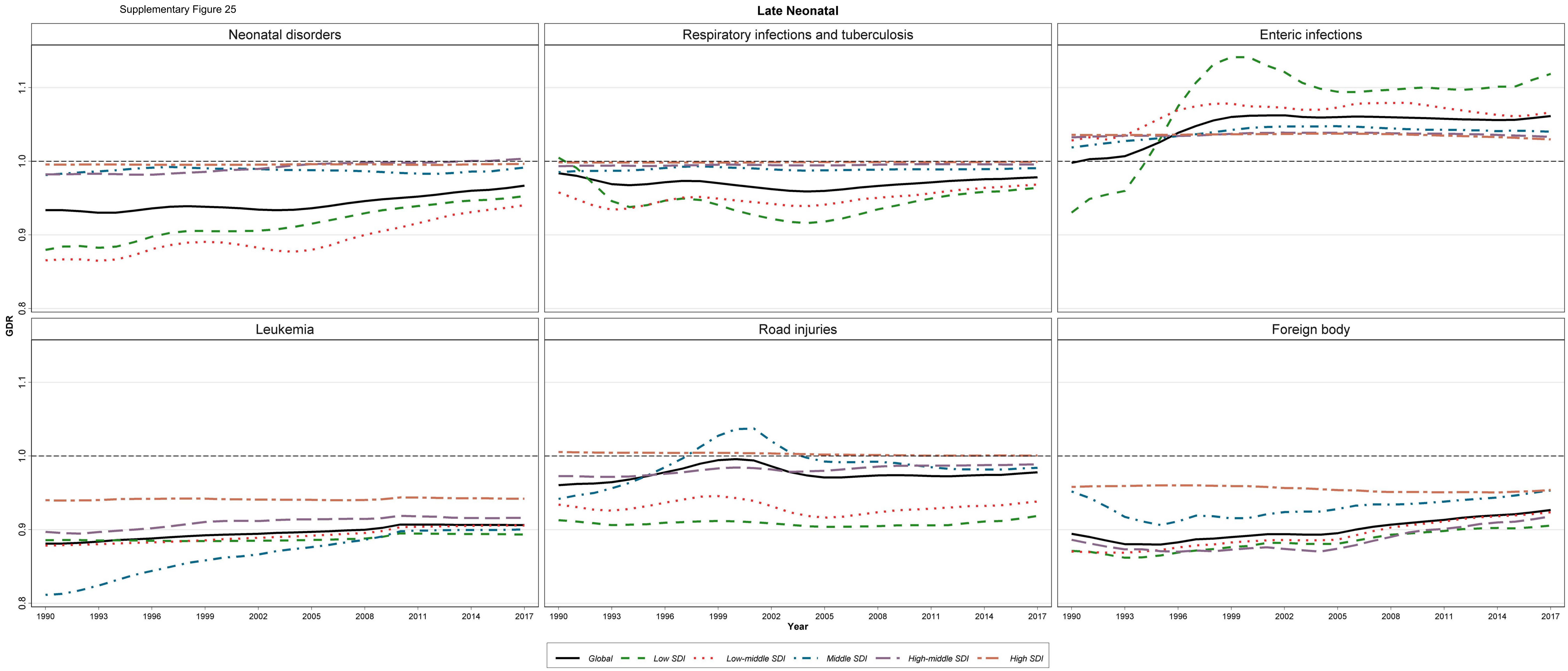

Supplementary Figure 26

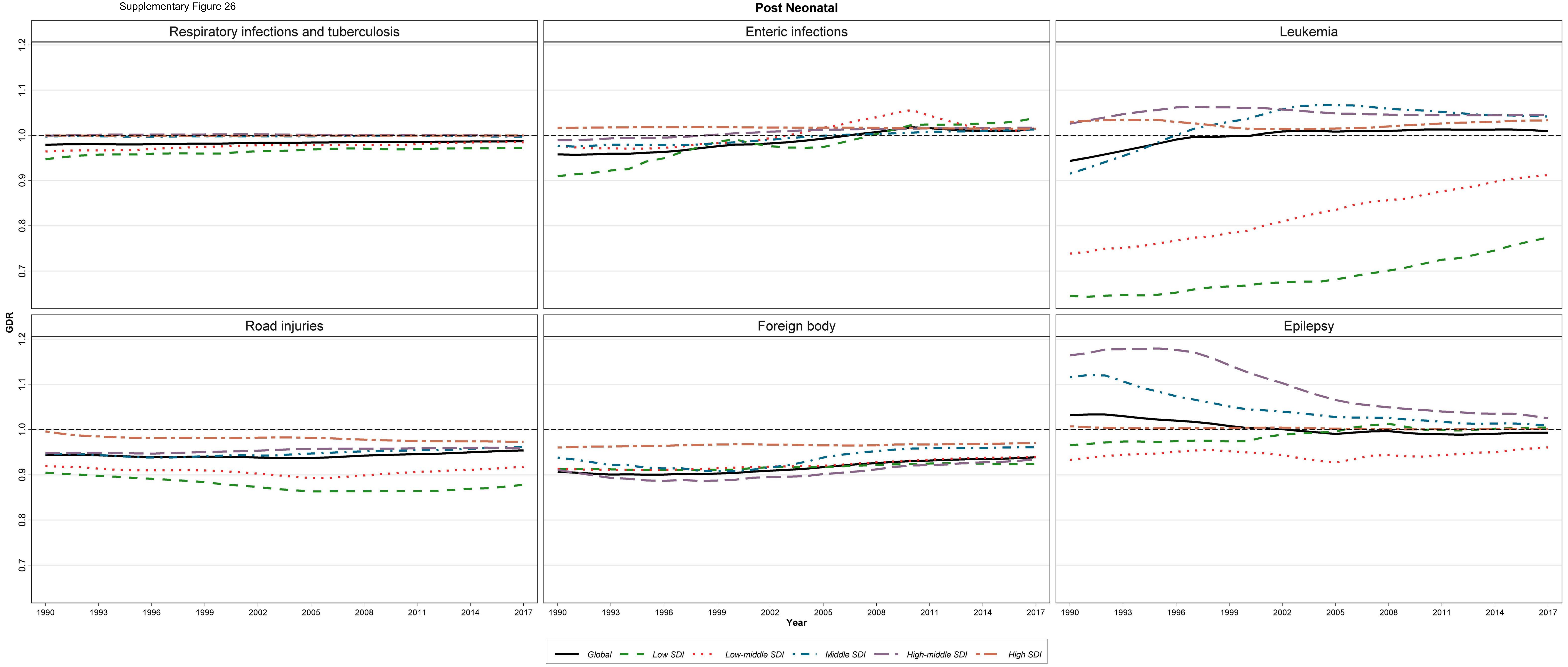

Supplementary Figure 27

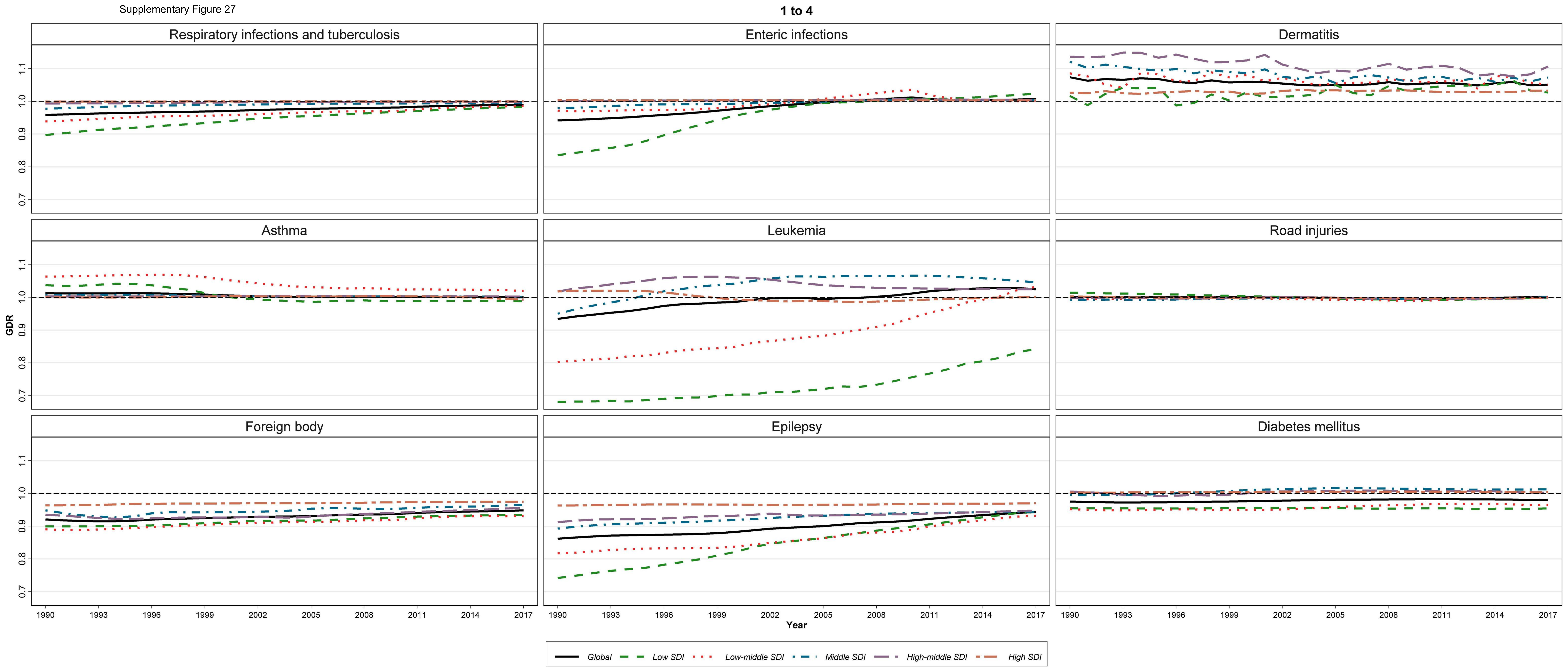

Supplementary Figure 28

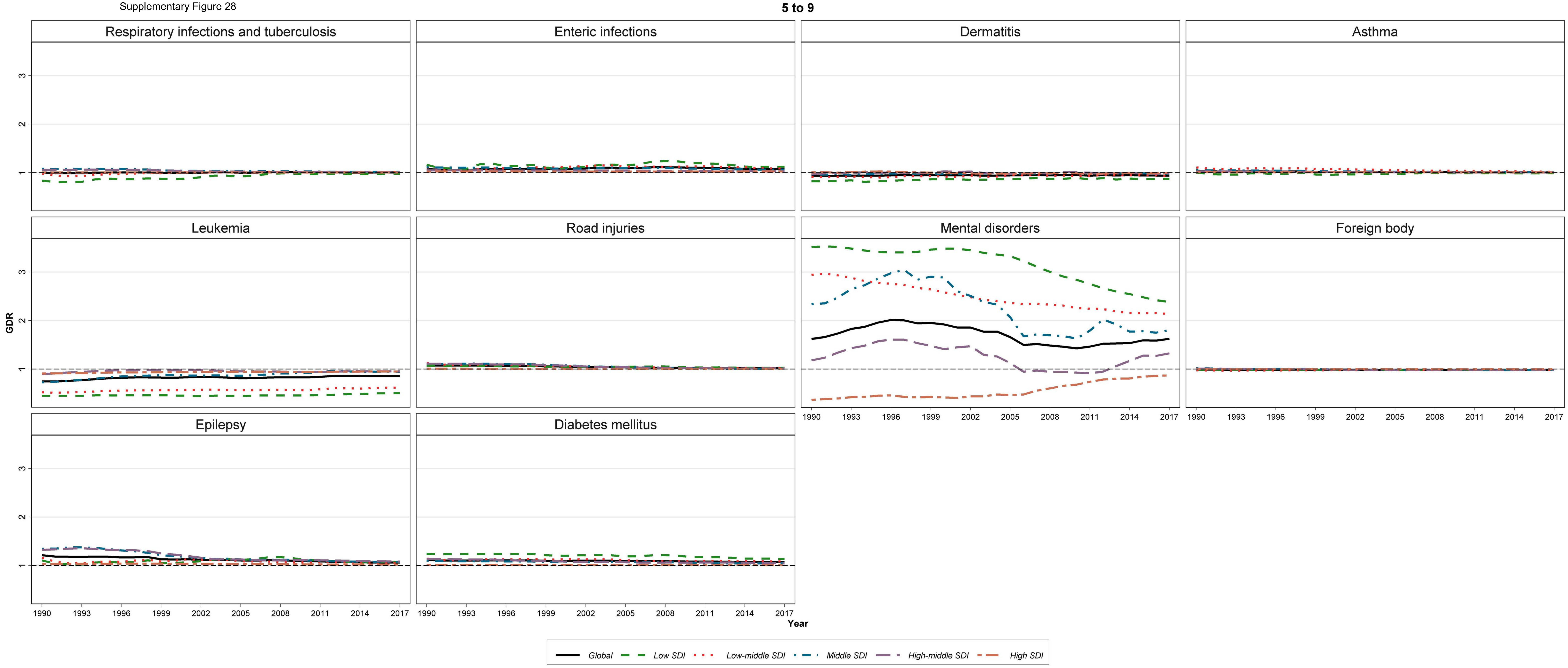

Supplementary Figure 29

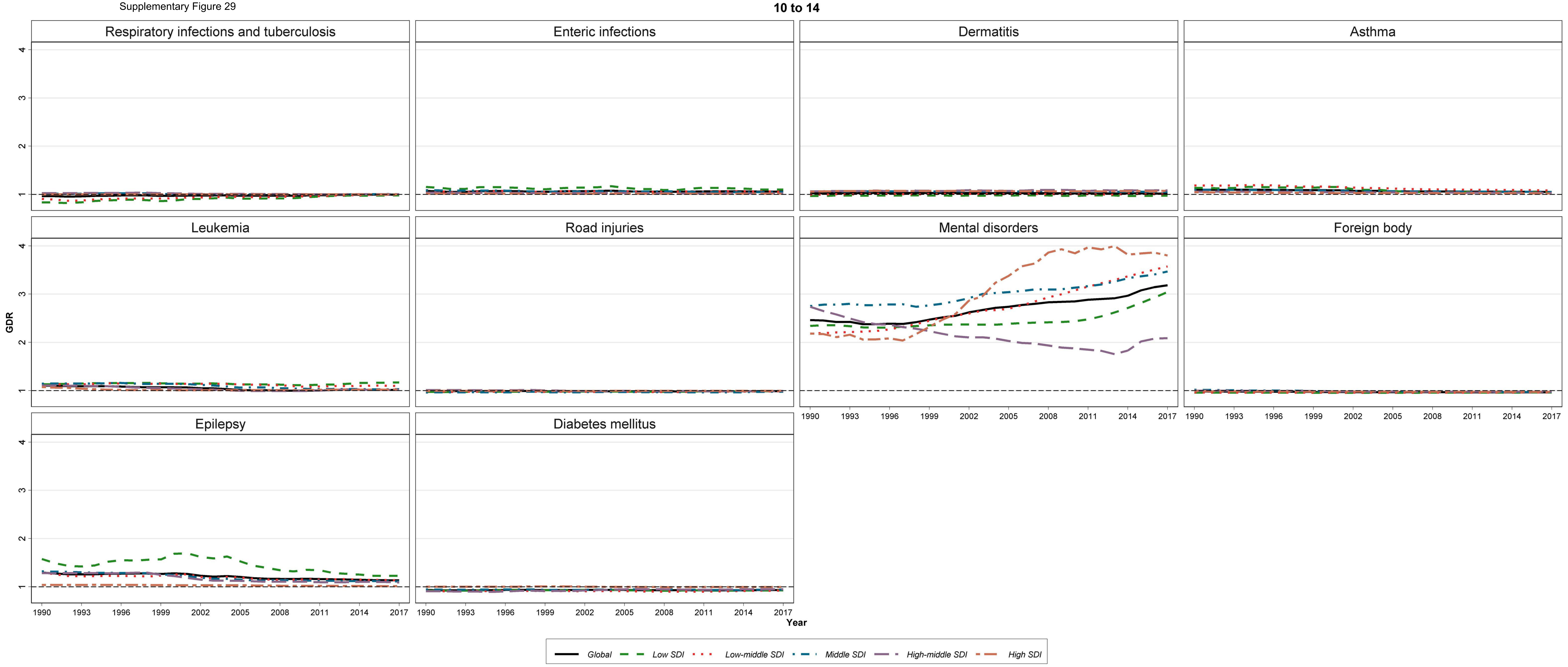

Supplementary Figure 30

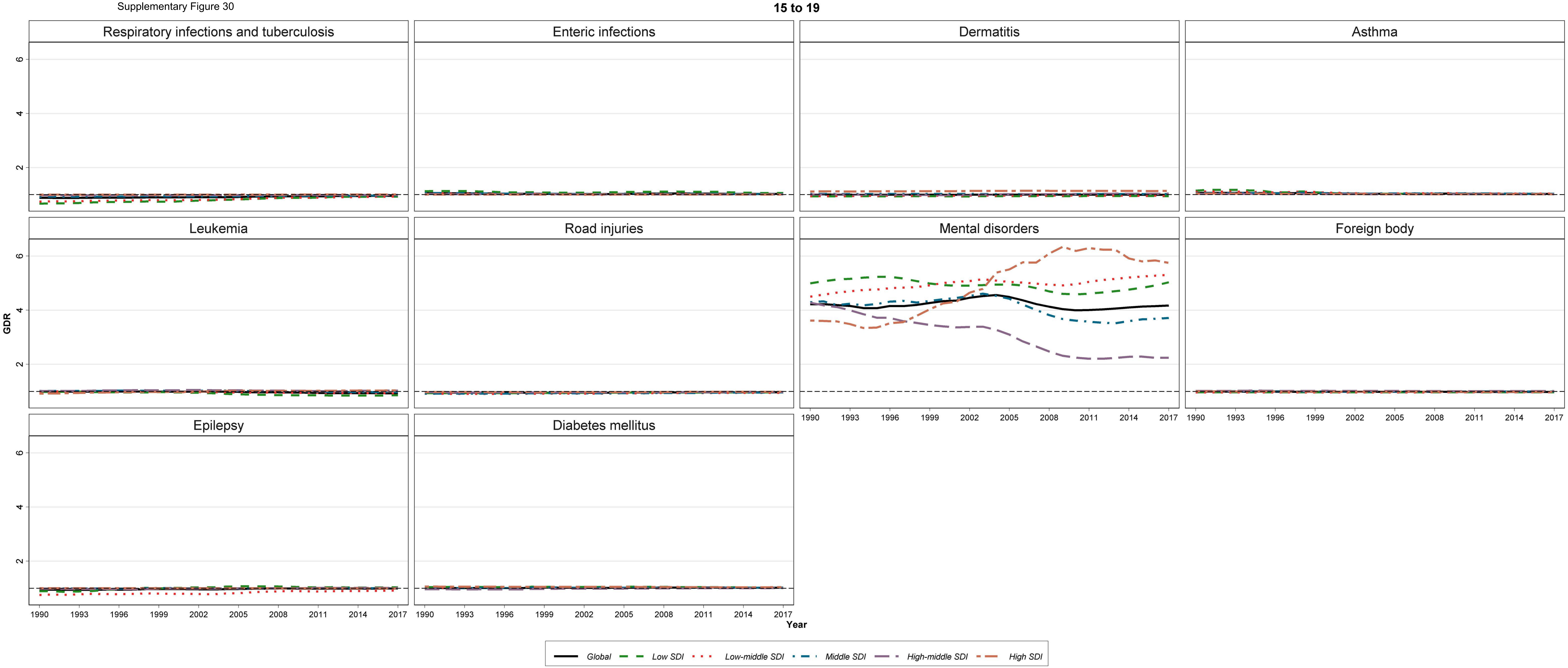

Both, 2017

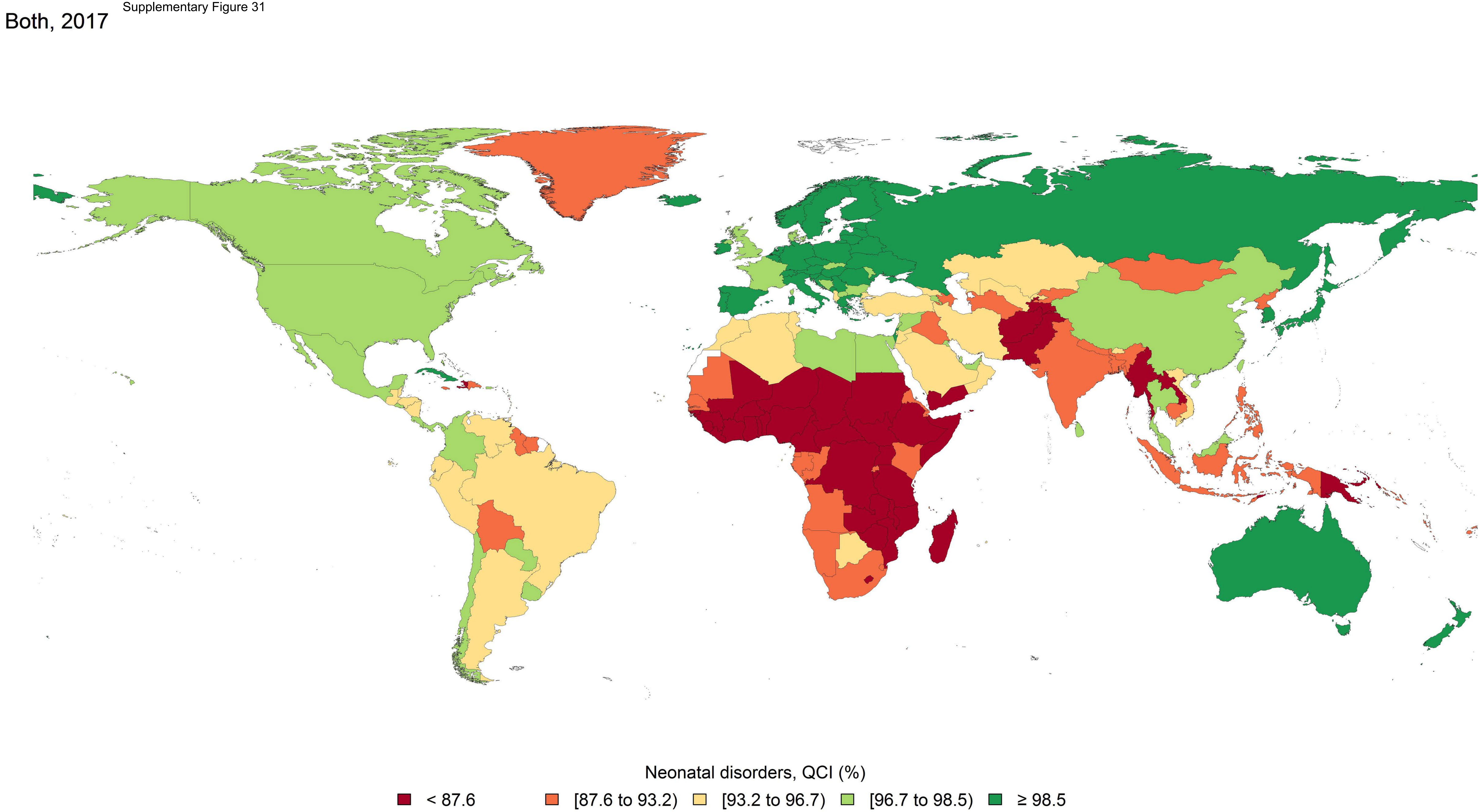
Neonatal disorders, QCI (%)
< 87.6
[87.6 to 93.2)
[93.2 to 96.7)
[96.7 to 98.5)
≥ 98.5

Both, 2017

Supplementary Figure 32

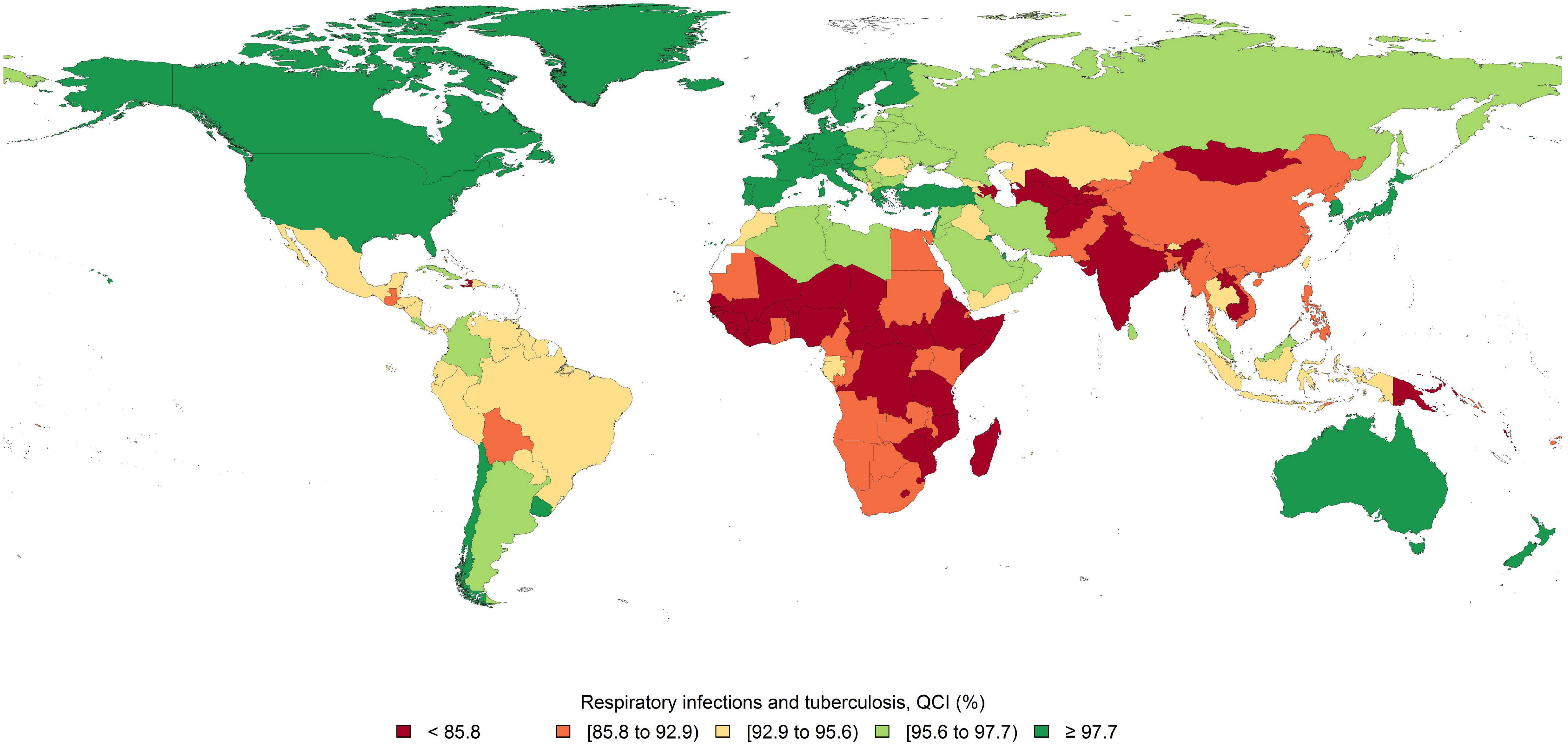

Supplementary Figure 33

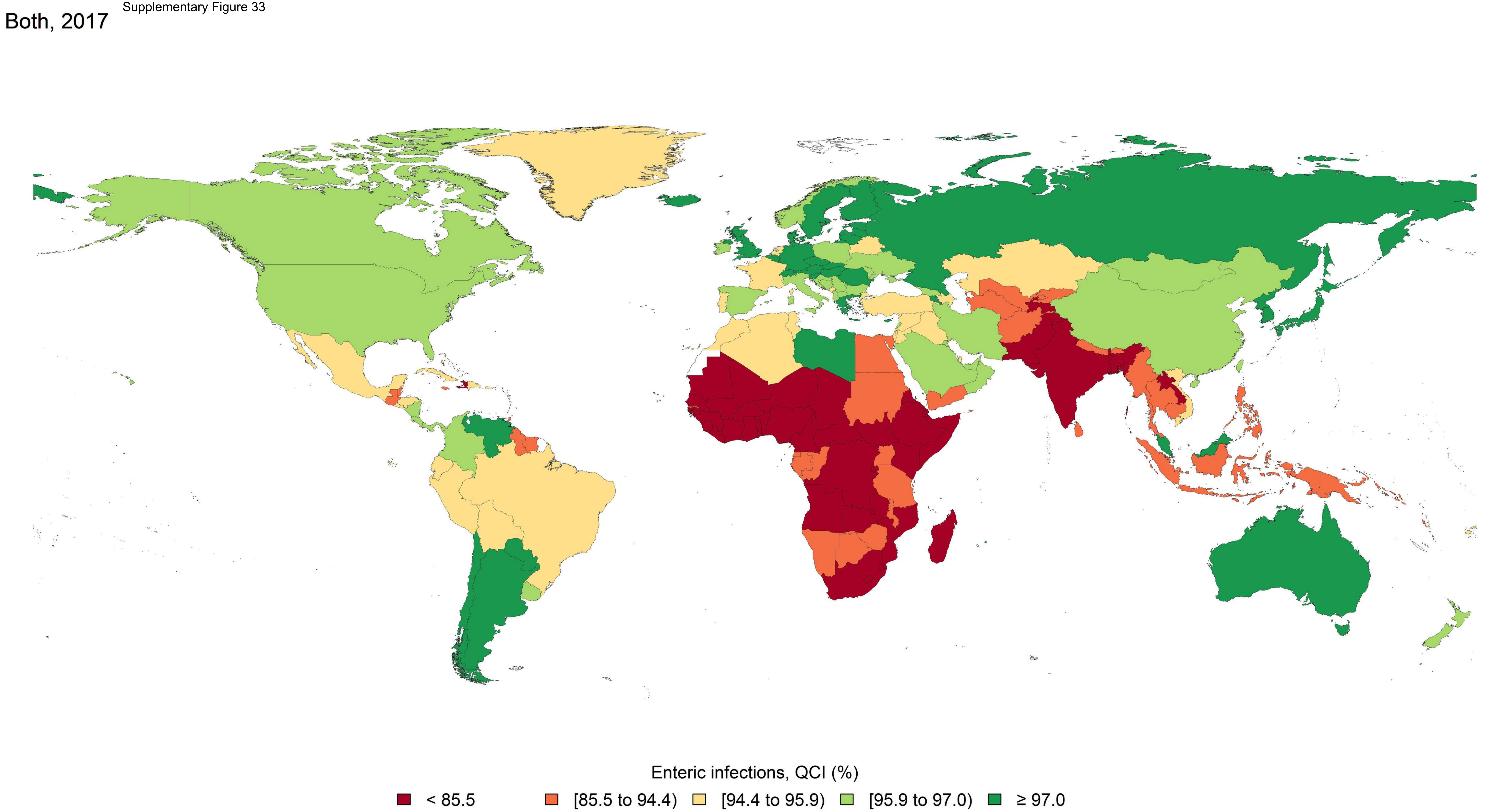

Supplementary Figure 34

Both, 2017

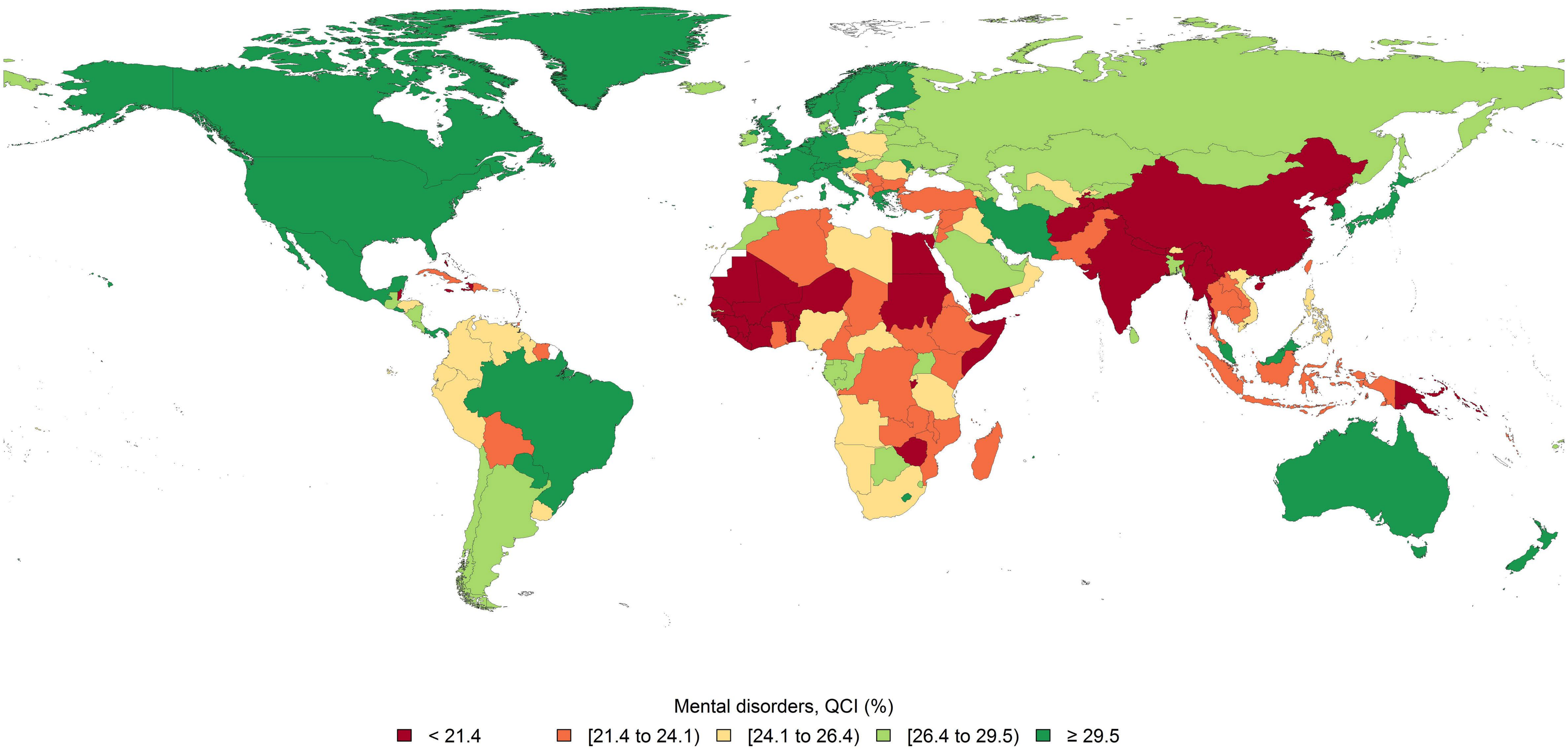

Supplementary Figure 35

Both, 2017

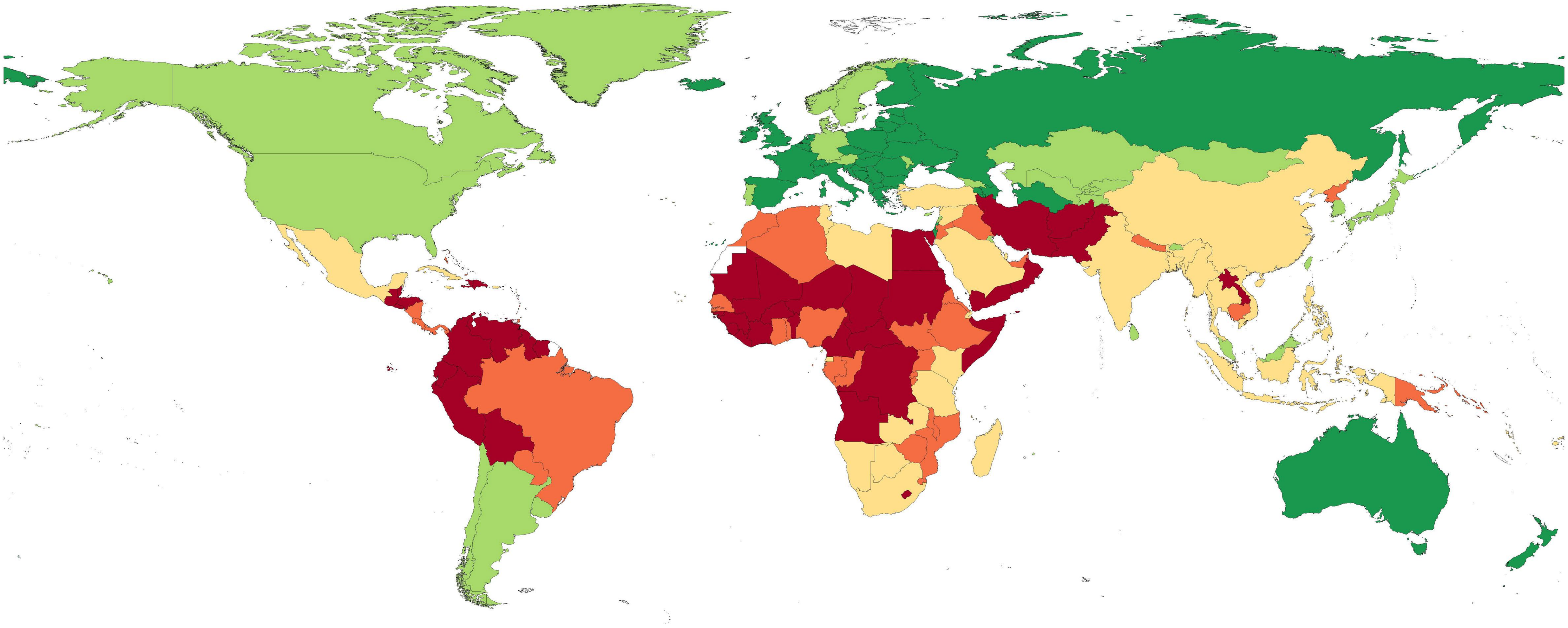

Road injuries, QCI (%)

< 86.4 | [86.4 to 89.5) | [89.5 to 93.6) | [93.6 to 98.1) | ≥ 98.1

Both, 2017

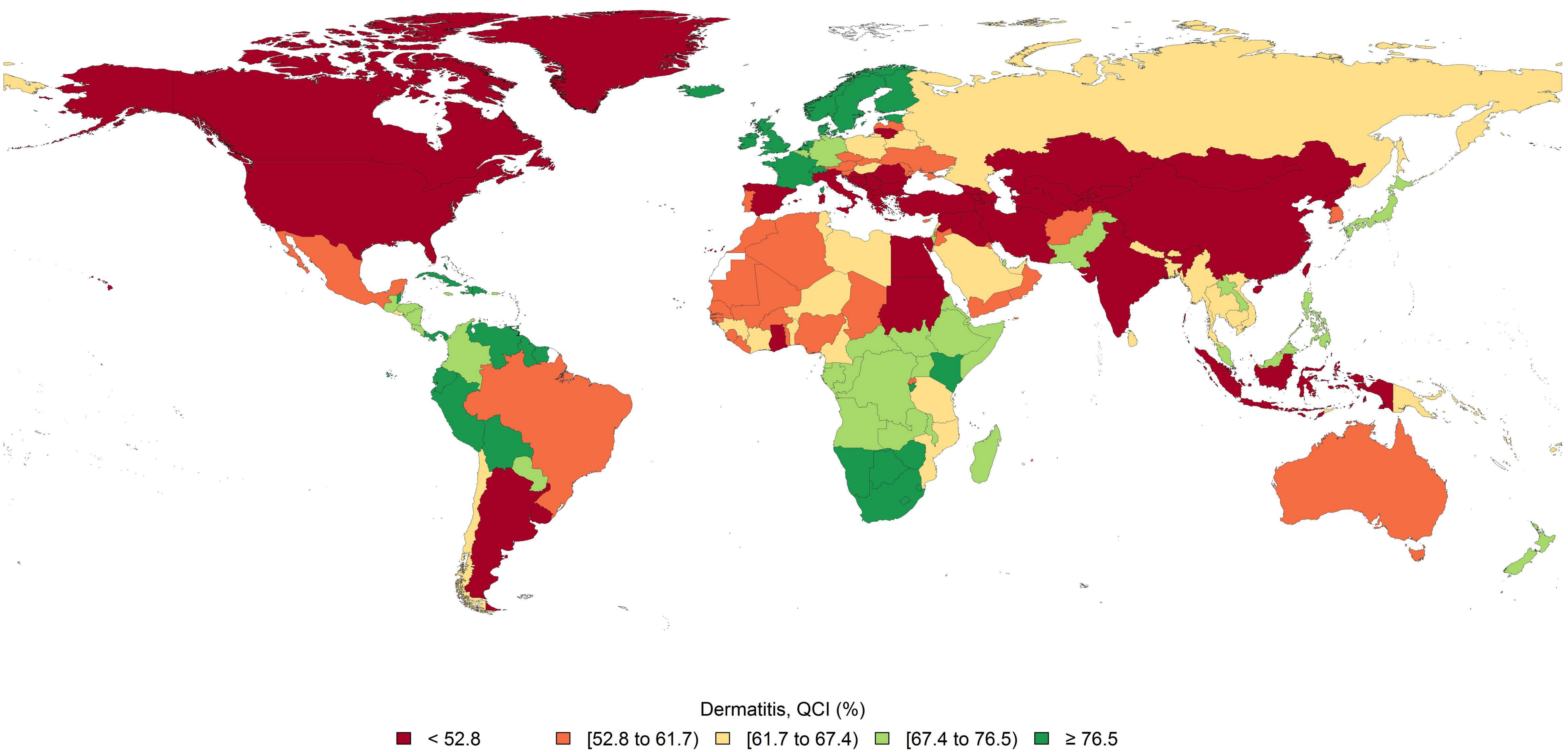

Supplementary Figure 37

Both, 2017

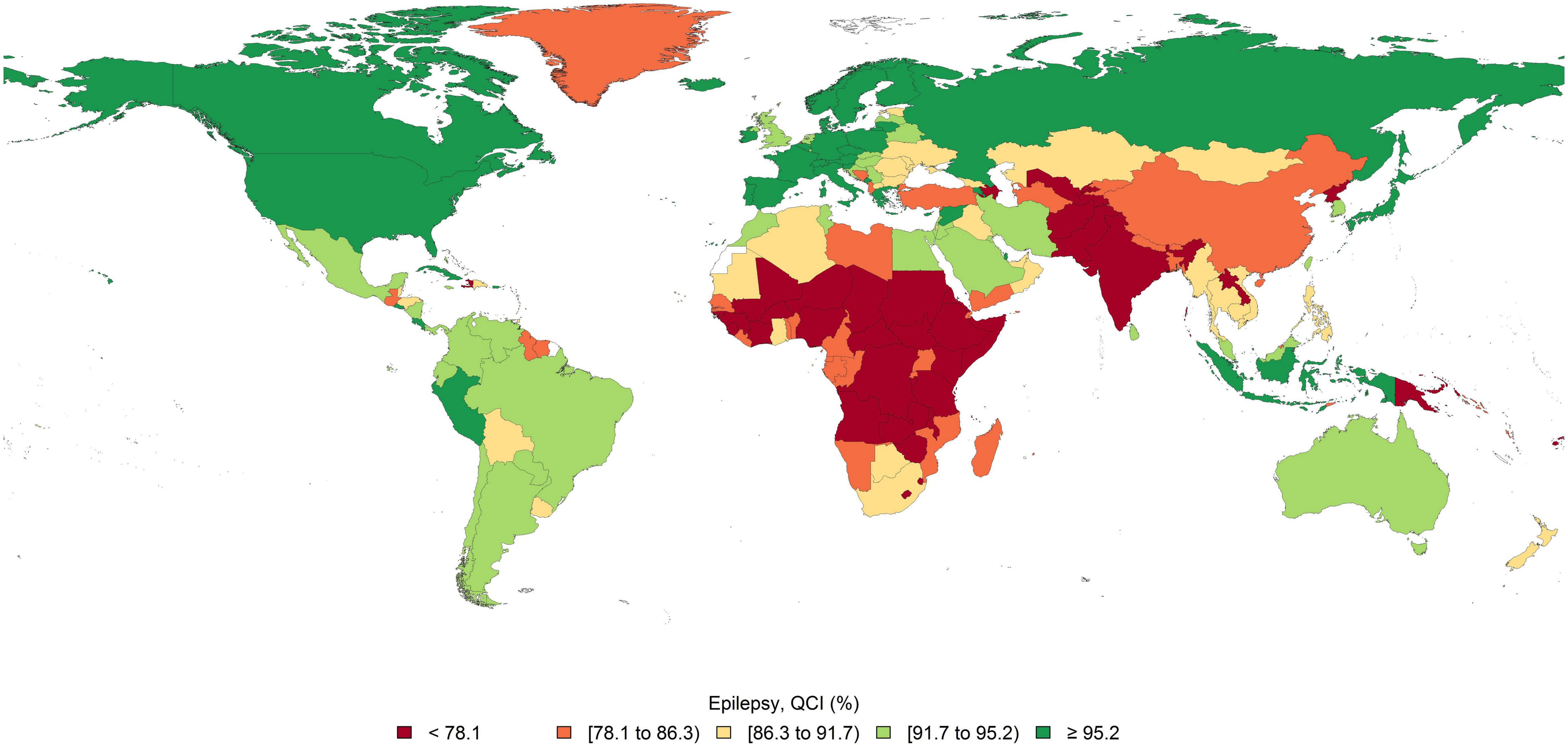

Supplementary Figure 38

Both, 2017

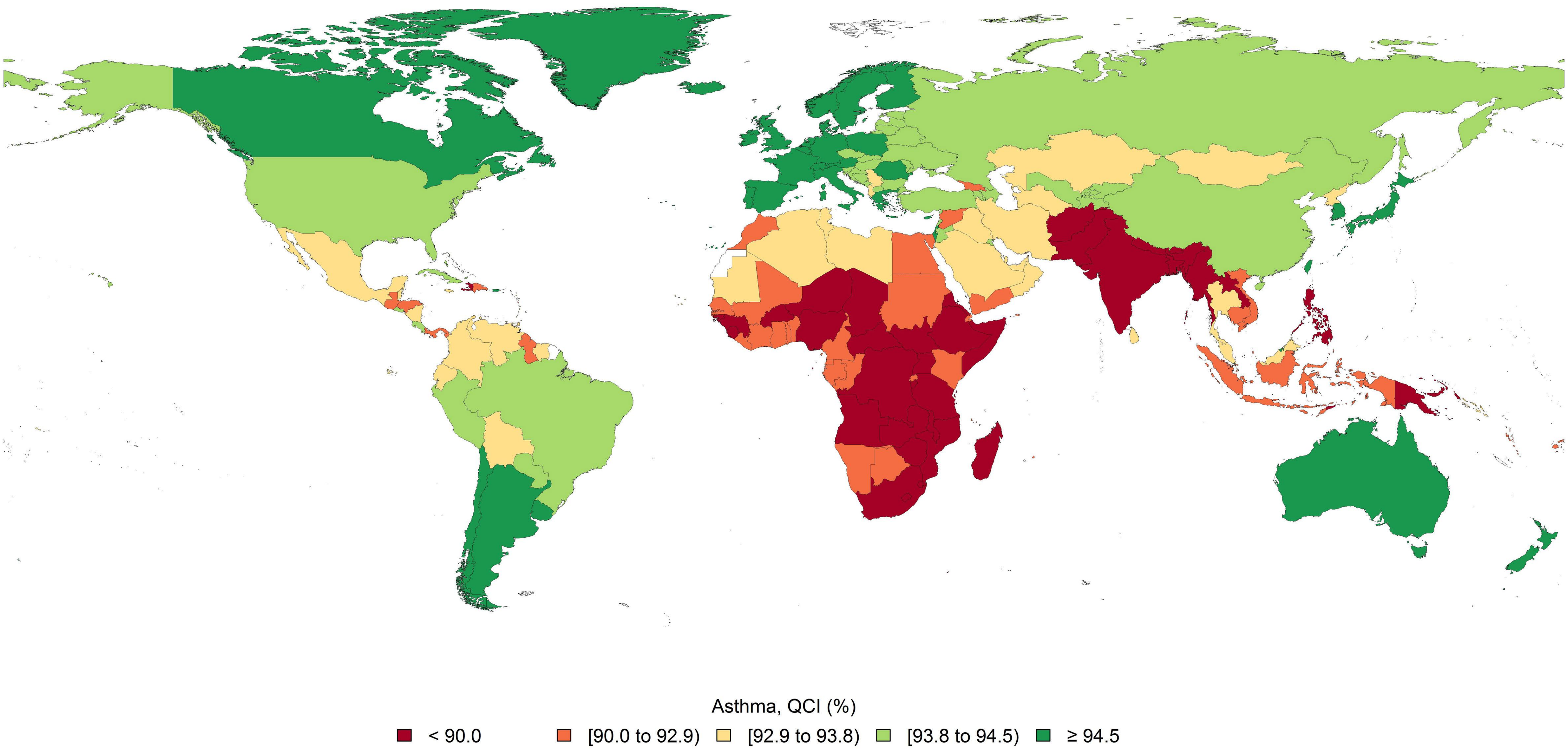

Supplementary Figure 39

Both, 2017

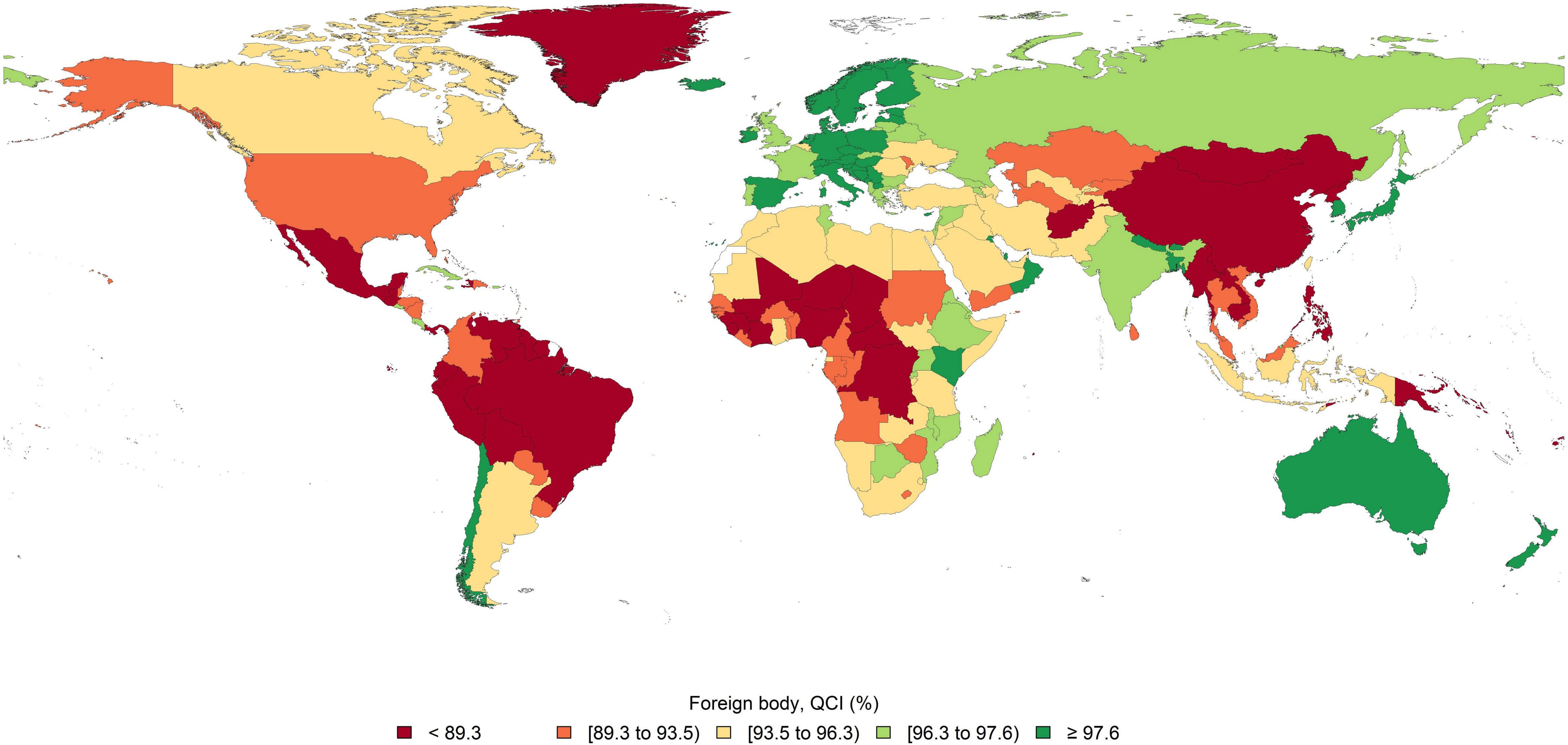

Supplementary Figure 40

Both, 2017

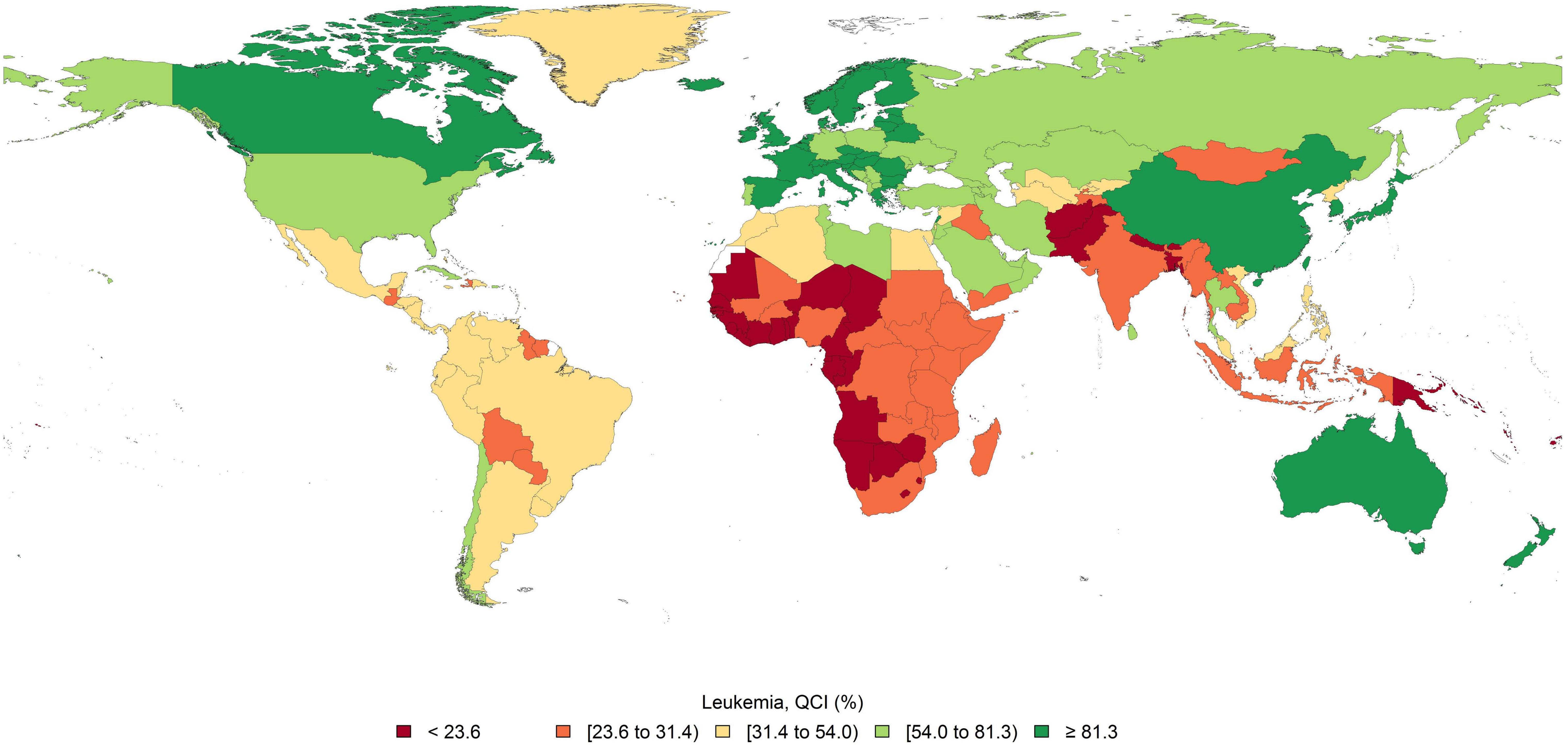

Supplementary Figure 41

Both, 2017

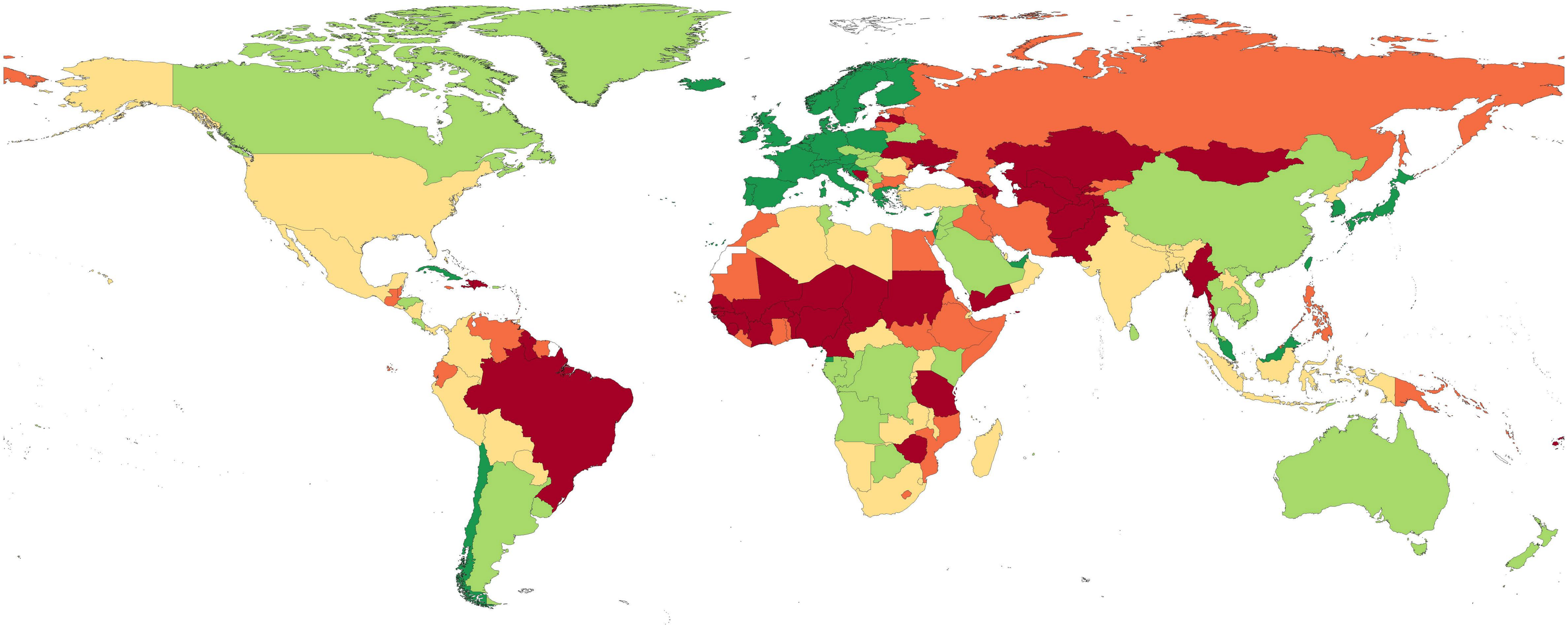

Diabetes mellitus, QCI (%)

< 69.2 | [69.2 to 78.6) | [78.6 to 84.2) | [84.2 to 91.7) | ≥ 91.7

Supplementary Figure 42

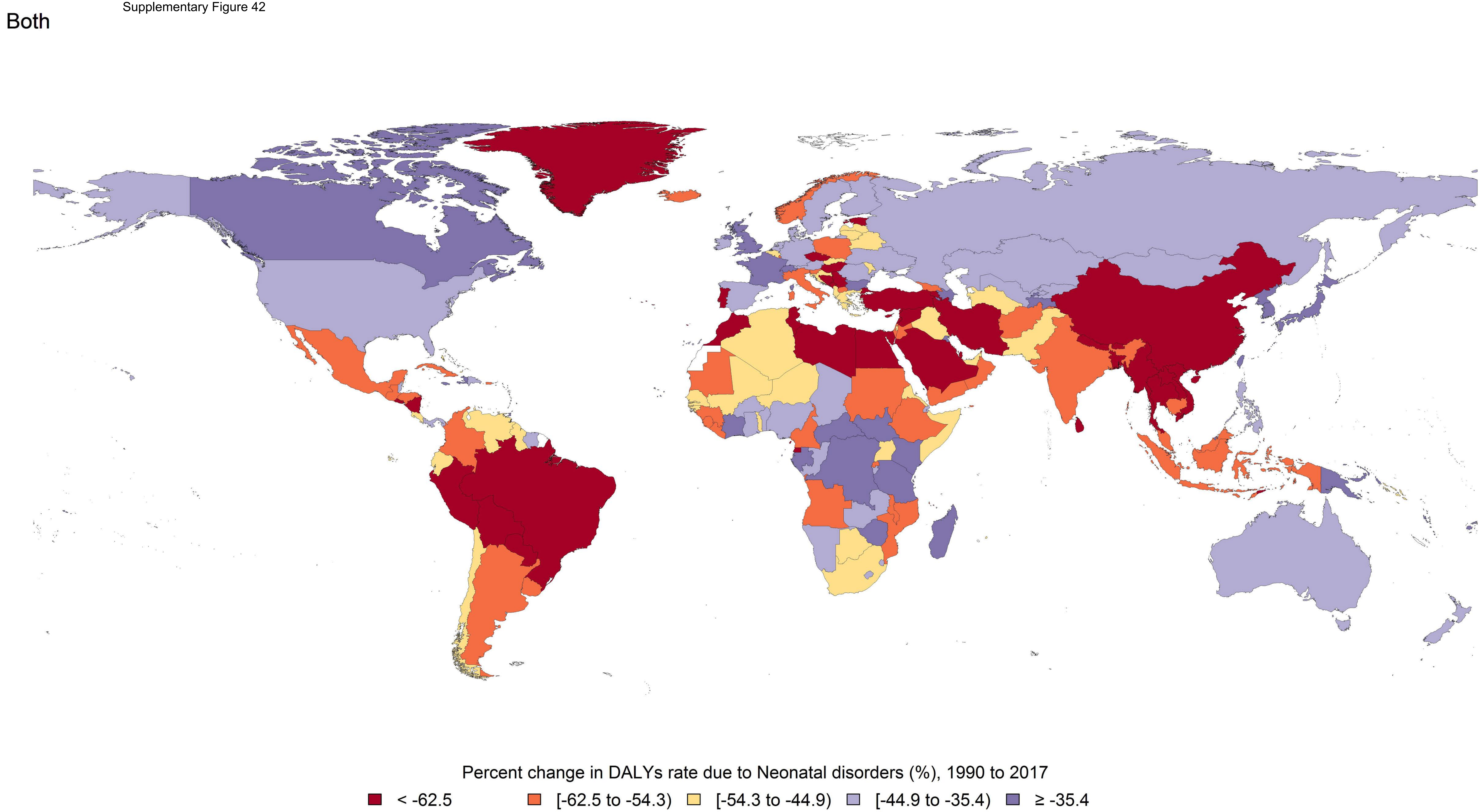

Both

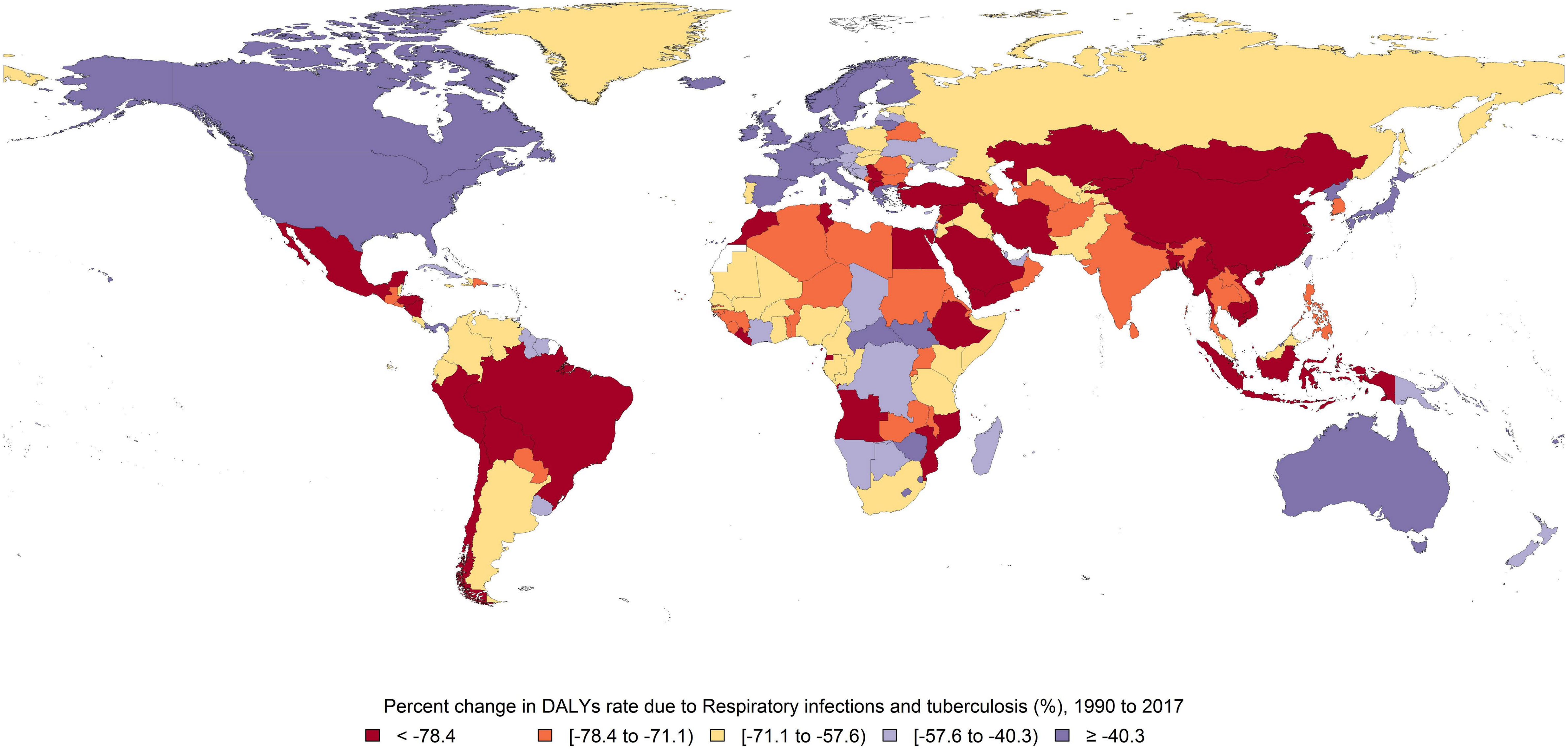
Percent change in DALYs rate due to Respiratory infections and tuberculosis (%), 1990 to 2017
< -78.4
[-78.4 to -71.1)
[-71.1 to -57.6)
[-57.6 to -40.3)
≥ -40.3

Supplementary Figure 44

Both

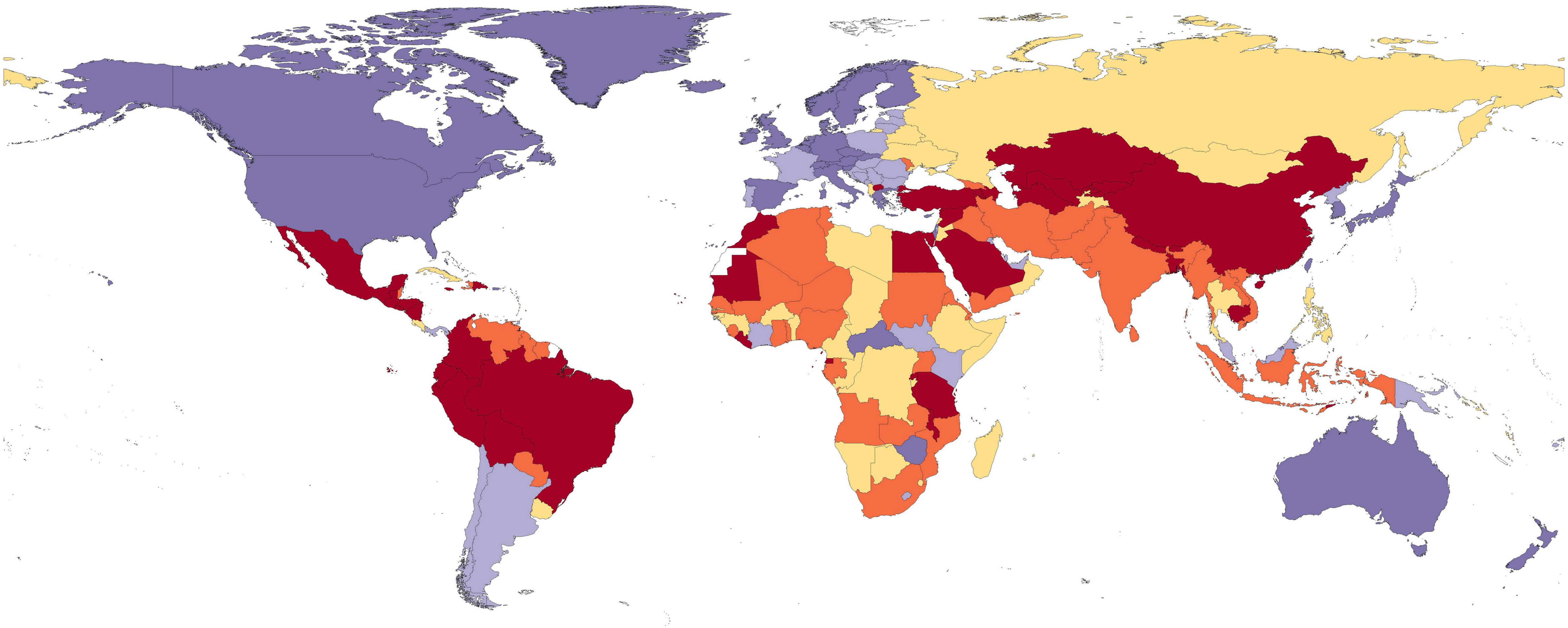

Percent change in DALYs rate due to Enteric infections (%), 1990 to 2017

< -81.2 | [-81.2 to -70.0) | [-70.0 to -48.3) | [-48.3 to -4.6) | ≥ -4.6

Supplementary Figure 45

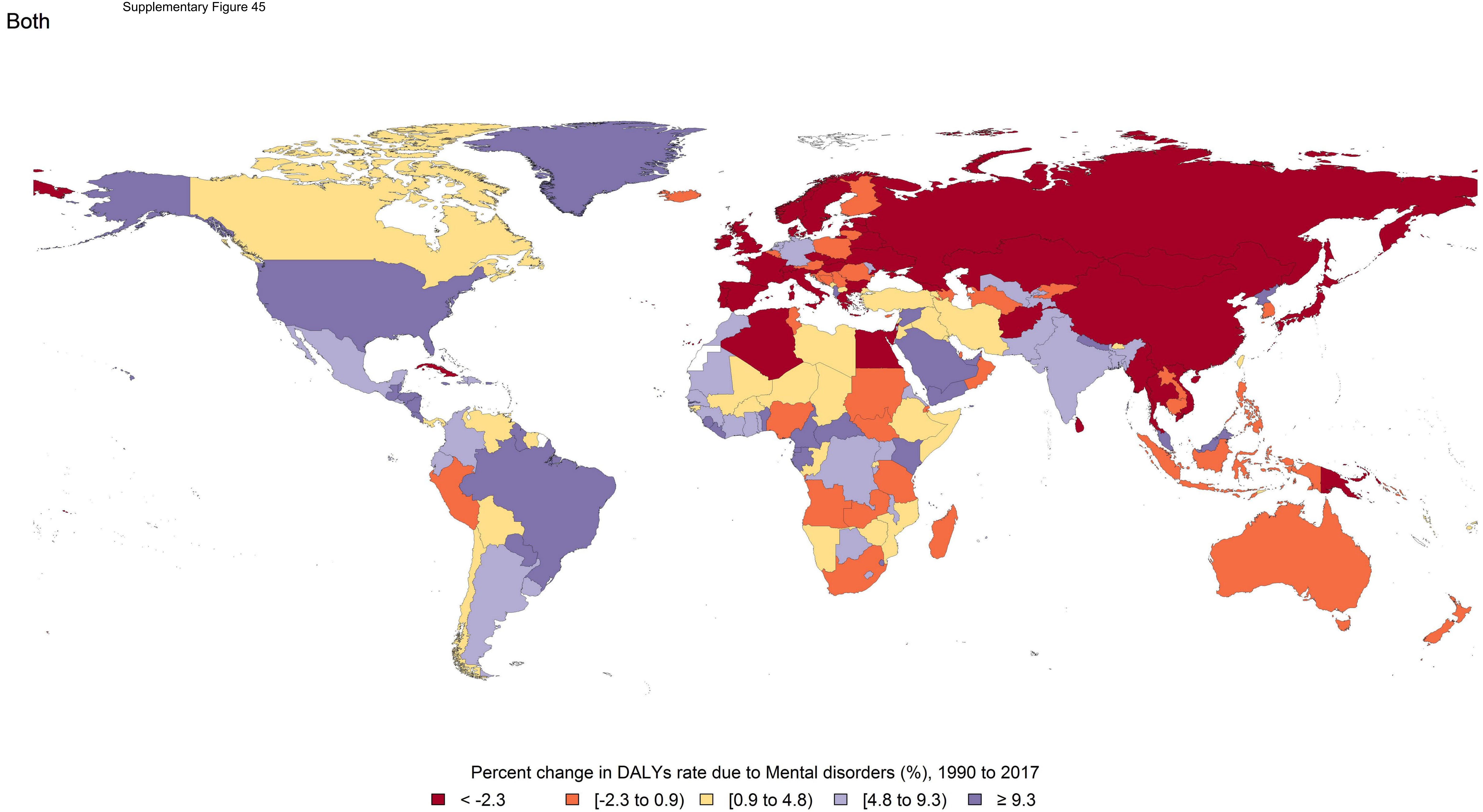

Supplementary Figure 46

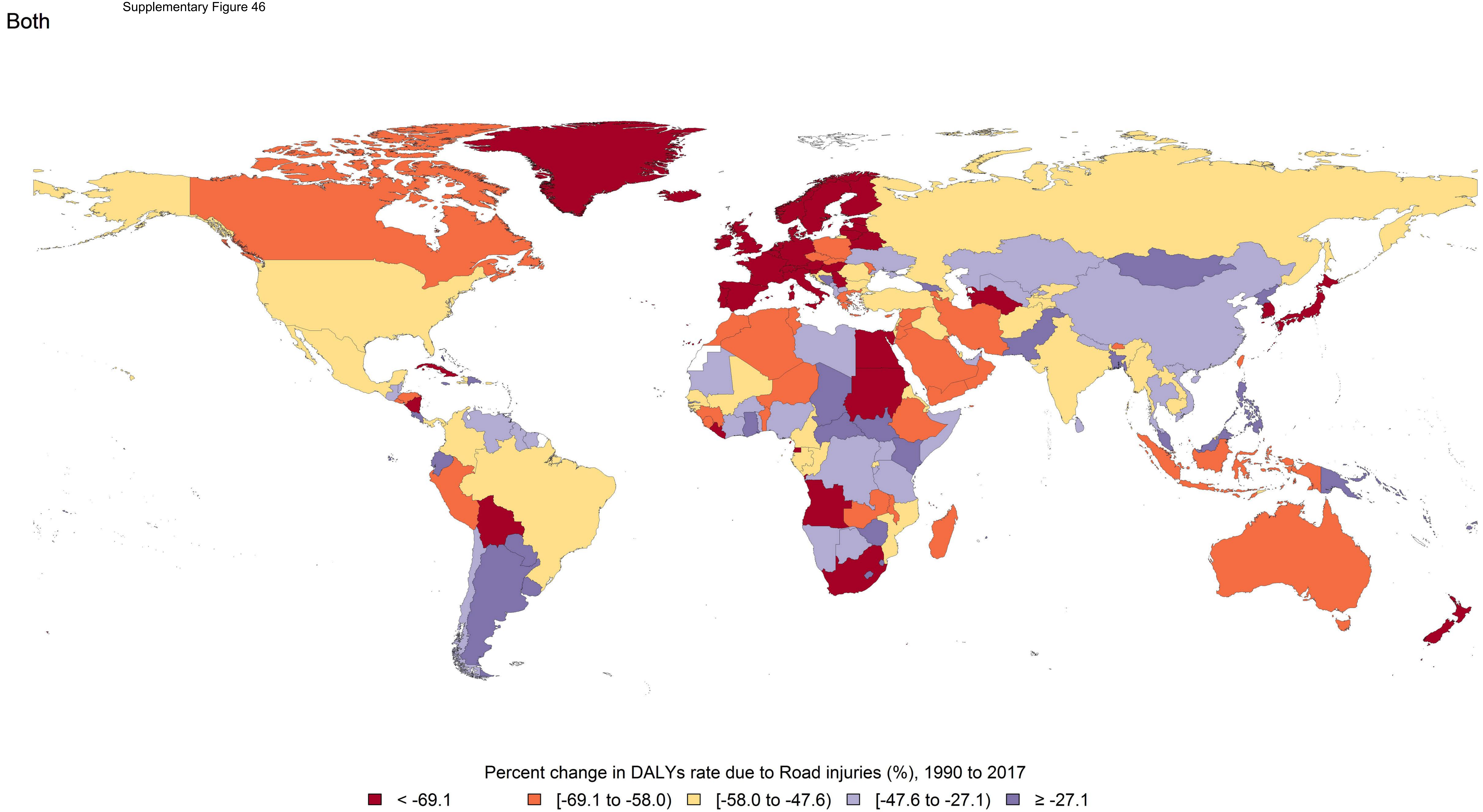

Both

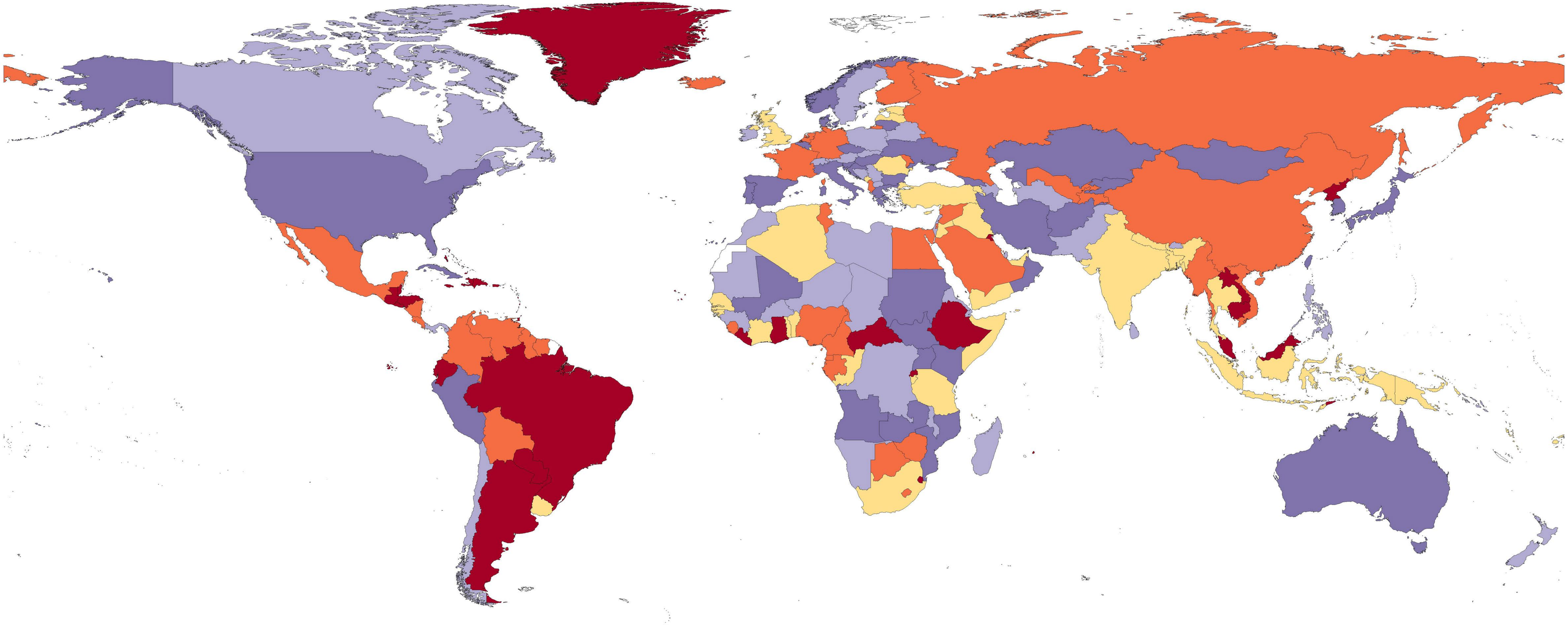

Percent change in DALYs rate due to Dermatitis (%), 1990 to 2017

< -1.5 | [-1.5 to -0.2) | [-0.2 to 0.4) | [0.4 to 1.5) | ≥ 1.5

Supplementary Figure 48

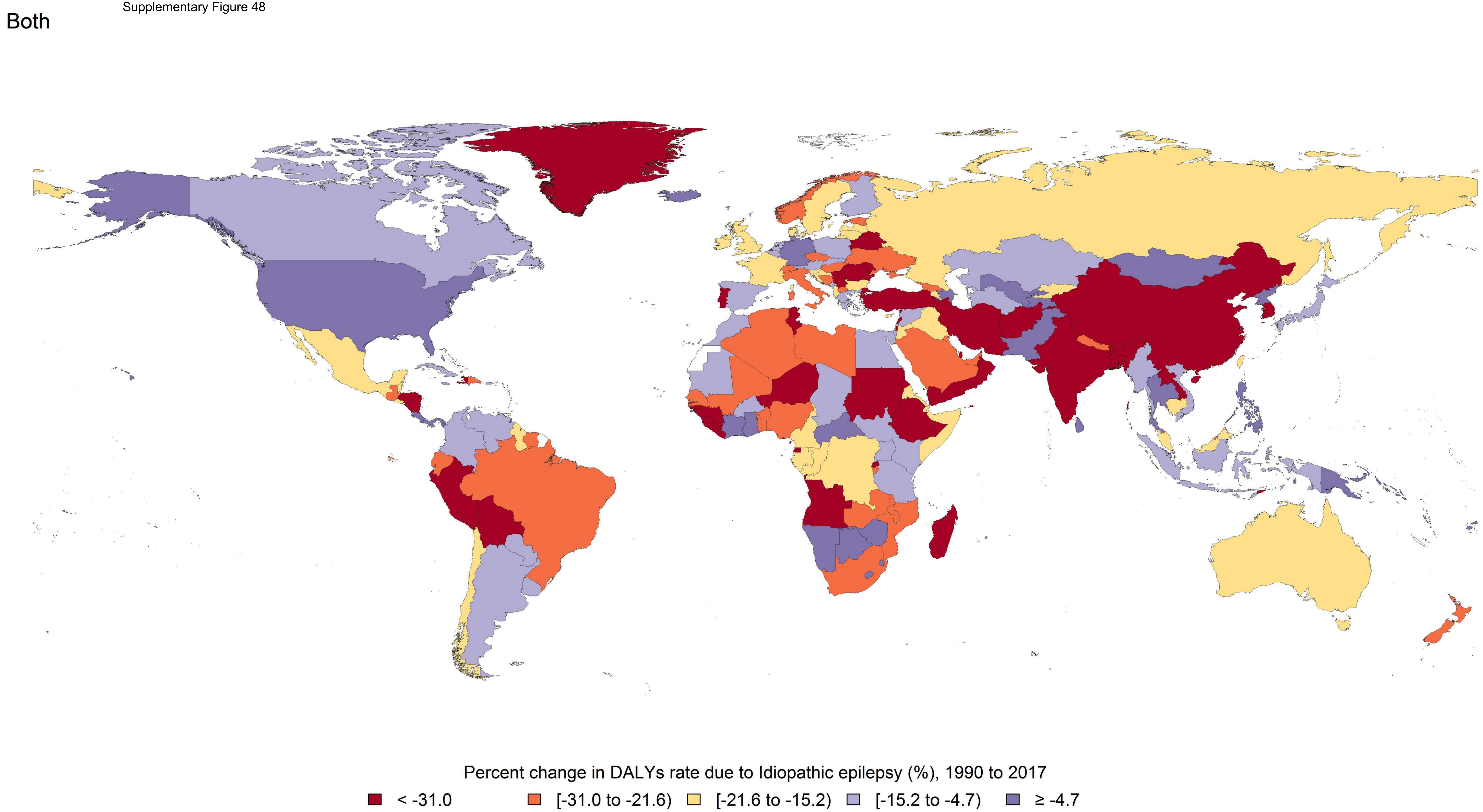

Supplementary Figure 49

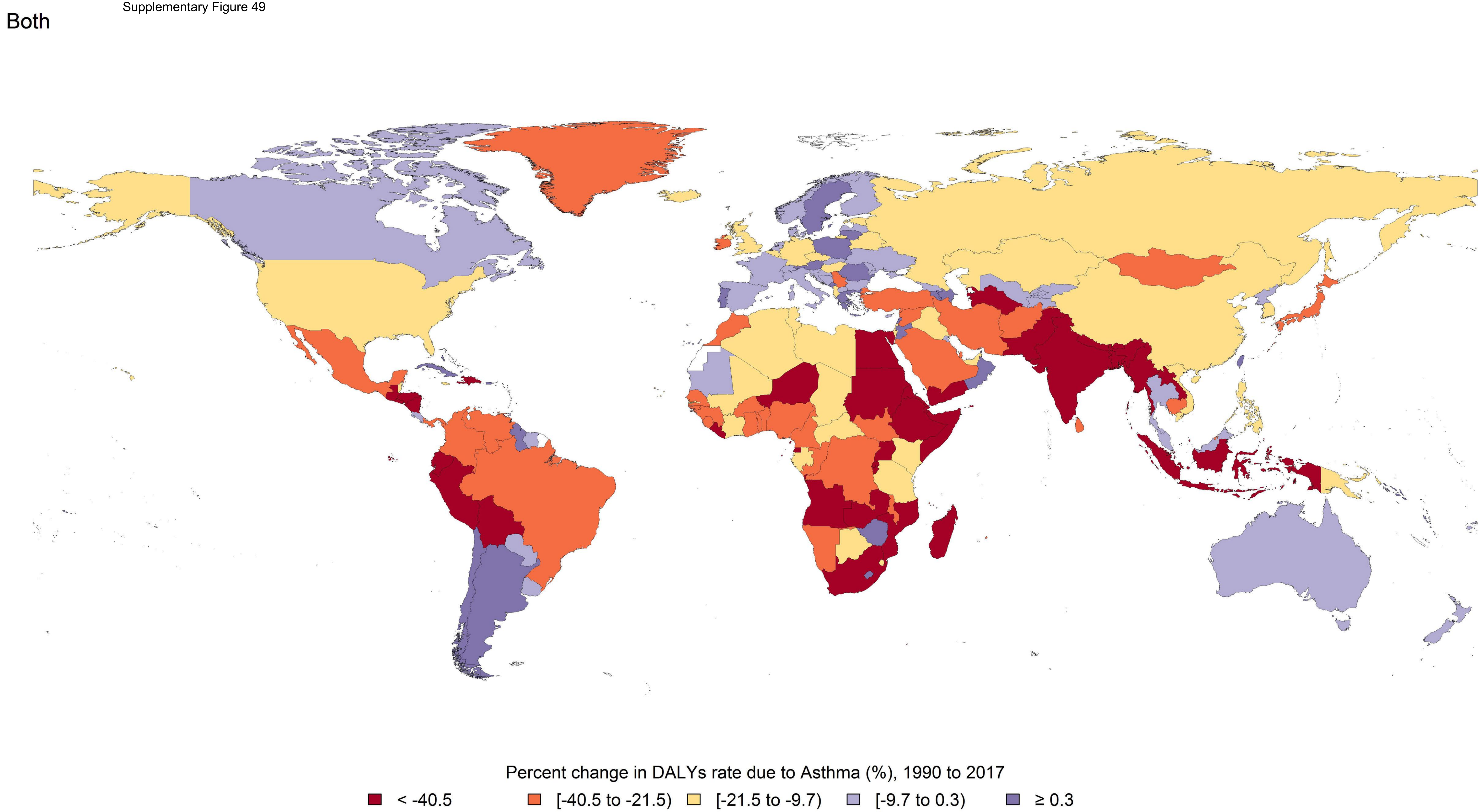

Both

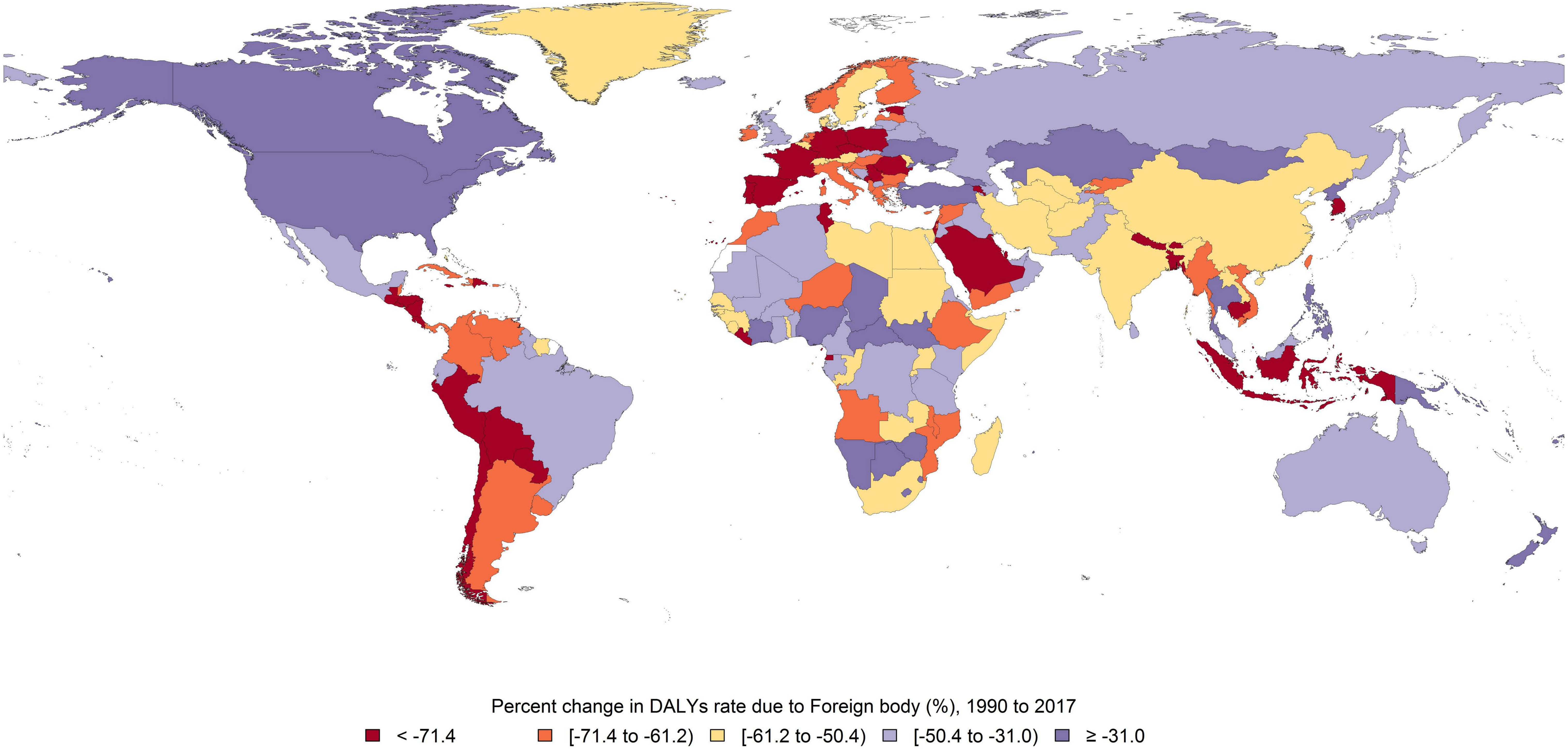
Percent change in DALYs rate due to Foreign body (%), 1990 to 2017
< -71.4
[-71.4 to -61.2)
[-61.2 to -50.4)
[-50.4 to -31.0)
≥ -31.0

Supplementary Figure 51

Both

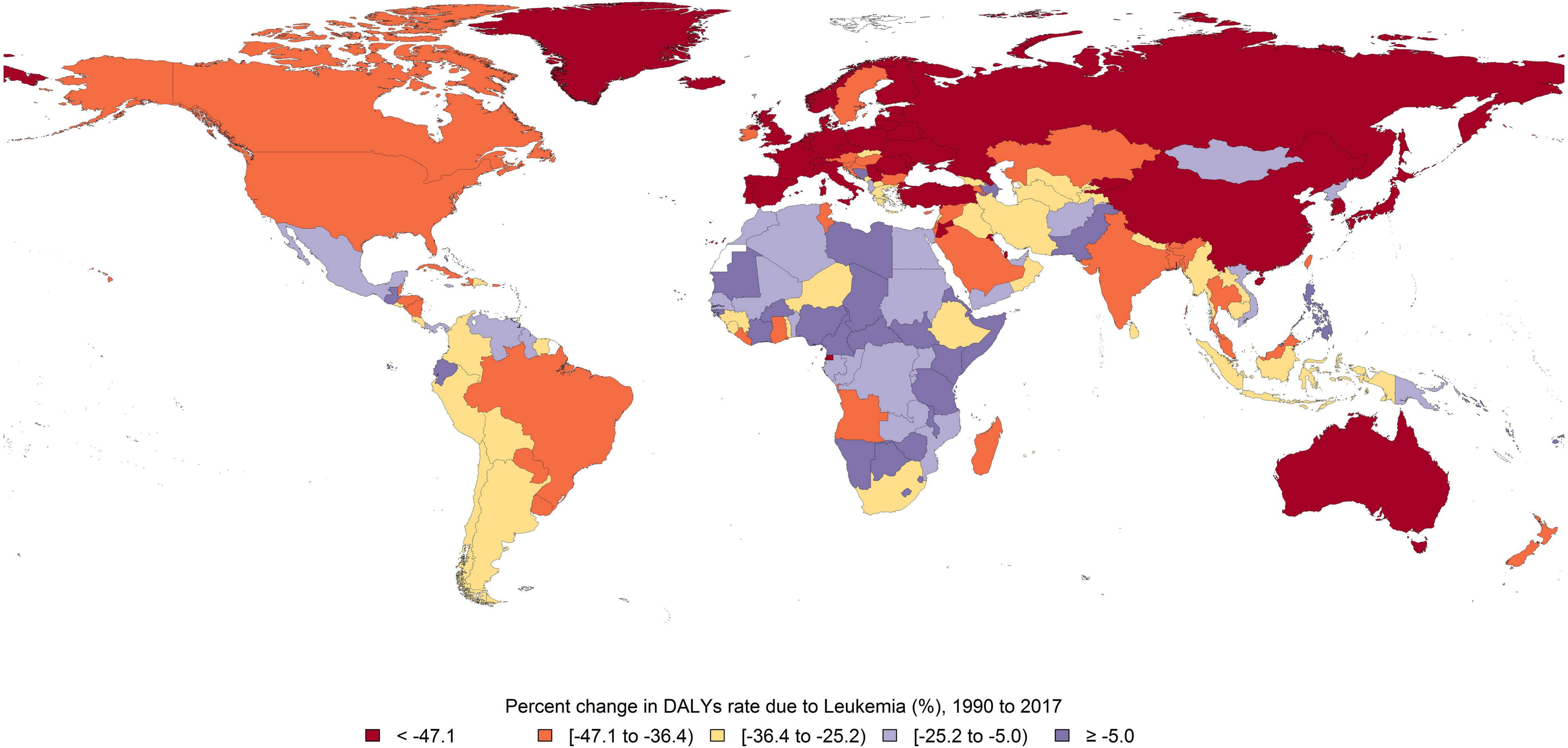

Supplementary Figure 52

Both

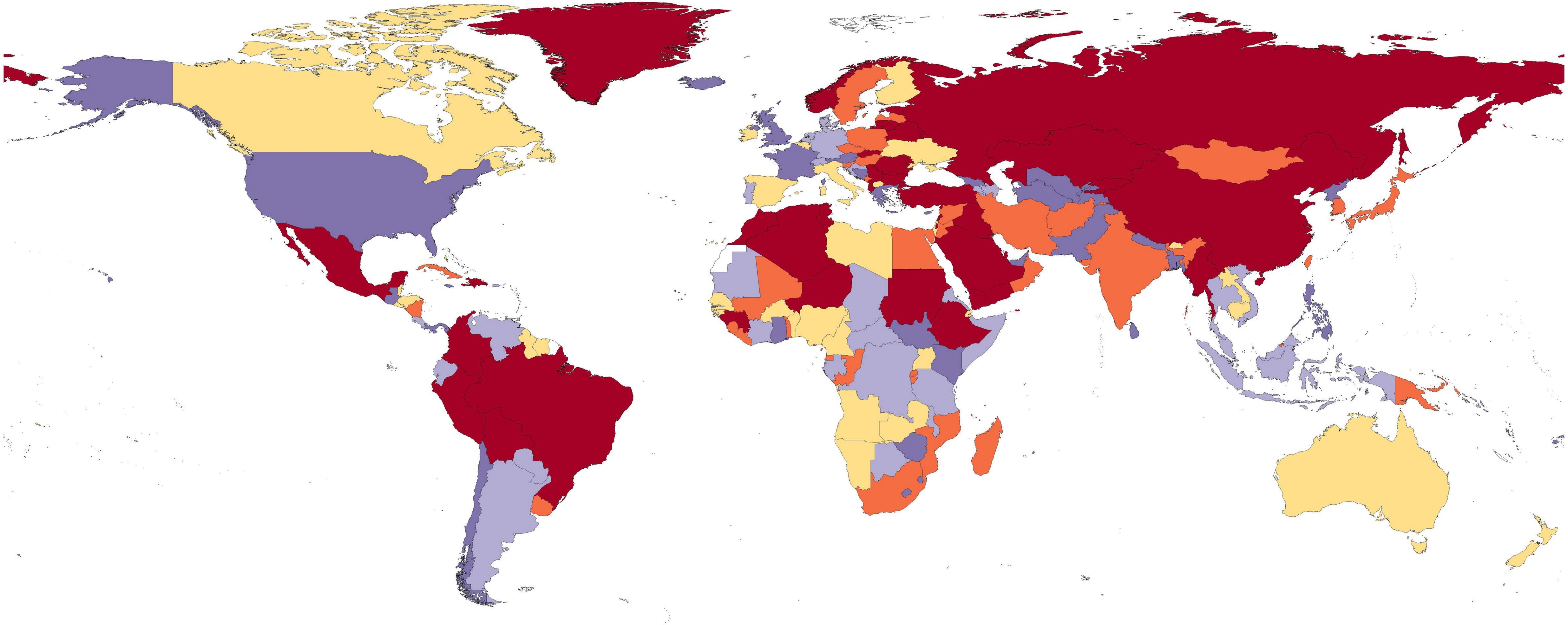

Percent change in DALYs rate due to Diabetes mellitus (%), 1990 to 2017

< -25.2 | [-25.2 to -13.6) | [-13.6 to -0.3) | [-0.3 to 13.3) | ≥ 13.3
